# Identifying differential expression in multiple SAGE libraries: an overdispersed log-linear model approach

**DOI:** 10.1186/1471-2105-6-165

**Published:** 2005-06-29

**Authors:** Jun Lu, John K Tomfohr, Thomas B Kepler

**Affiliations:** 1 Department of Biostatistics & Bioinformatics, Duke University, Durham, North Carolina 27708, USA

## Abstract

**Background:**

In testing for differential gene expression involving multiple serial analysis of gene expression (SAGE) libraries, it is critical to account for both between and within library variation. Several methods have been proposed, including the *t *test, *t*_*w *_test, and an overdispersed logistic regression approach. The merits of these tests, however, have not been fully evaluated. Questions still remain on whether further improvements can be made.

**Results:**

In this article, we introduce an overdispersed log-linear model approach to analyzing SAGE; we evaluate and compare its performance with three other tests: the two-sample *t *test, *t*_*w *_test and another based on overdispersed logistic linear regression. Analysis of simulated and real datasets show that both the log-linear and logistic overdispersion methods generally perform better than the *t *and *t*_*w *_tests; the log-linear method is further found to have better performance than the logistic method, showing equal or higher statistical power over a range of parameter values and with different data distributions.

**Conclusion:**

Overdispersed log-linear models provide an attractive and reliable framework for analyzing SAGE experiments involving multiple libraries. For convenience, the implementation of this method is available through a user-friendly web-interface available at .

## Background

Serial analysis of gene expression (SAGE) is used to measure relative abundances of messenger RNAs (mRNAs) for a large number of genes [1,2]. Briefly, mRNAs are extracted from biological samples and reverse-transcribed to cDNAs. The double-stranded cDNAs are then digested by a 4-cutter restriction enzyme (anchoring enzymes, usually NlaIII). After digestion, another restriction enzyme (tagging enzymes) is used to release the downstream DNA sequences at 3' of most of the anchoring enzyme restriction sites. The released sequences, usually 10–11 base pairs (bp) long, are called SAGE tags. The tags derived from many different species of mRNAs can be concatenated, cloned and sequenced. In a typical SAGE experiment, a large number of tags (often ranging from 30,000 to 100,000) are collected from each sample, with each tag representing, ideally, one gene; the tag count indicates the transcription level of the gene represented by that specific tag. A natural question of interest is whether a given tag is differentially expressed. Over the past few years, SAGE has been extensively used for expression analysis of cancer samples for identifying diagnostic or therapeutic targets [3,4].

Most SAGE studies focus on comparing expression levels between two samples. For such two-library comparisons, several statistical methods have been proposed, such as the simulation method of Zhang *et al. *[2], the Bayesian approaches [5-7], and the normal approximation based *z*-test [8] (which is equivalent to the chi-square test [9]). A comparative review by Ruijter *et al. *[10] has shown that all these methods perform equally well.

The comparison between two SAGE libraries can identify biologically interesting tags (or genes). However, in many cases it is essential to conduct experiments with replicates in order to account for normal background biological variation. For experiments involving multiple SAGE libraries, between-library variation beyond the binomial sampling variation is introduced. Such between-library variation can be due to additional known factors involved in the experimental design, as well as to unknown genetic or environmental variation between observations. Indeed, major differences in gene expression exist among SAGE libraries prepared from the same tissues of different individuals [11]. Statistical methods are needed for analyzing SAGE experiments involving multiple libraries. In the case of two-group comparisons (e.g. comparisons between a normal group and a cancer group), methods such as pooling the libraries in each group and transforming to two-library comparisons (for example, using the chi-square test), or the two-sample *t-*test on proportions have been proposed and discussed [12-14]. The pooling approach is often problematic since it ignores gene expression variation among libraries within the same treatment group, which leads to biased estimates for the variance. The two-sample *t-*test on proportions, however, can be problematic as well; proportions estimated from libraries with smaller sizes are known to be more variable than those from larger libraries.

For two-group comparisons, Baggerly *et al. *introduced a test statistic, *t*_*w*_, based on a hierarchical beta-binomial model to account for both between-library and within-library variation [13]. The *t*_*w *_test statistic is assumed to have an approximate *t-*distribution and like the *t-*test, the *t*_*w*_-test is only good for two-group comparisons. For SAGE experiments with a more general design (e.g. involving 2 or more factors), an approach based on overdispersed logistic regression has been described [15]. Overdispersed models aim to allow for the possibility of overdispersion in the tag counts, i.e., cases where the variance in tag counts exceeds what is expected for binomial or Poisson sampling alone. Besides its flexibility in modeling multiple factors and/or continuous covariates, logistic regression compares group proportions on a logit scale (log of odds) rather than a raw scale as in the *t *and *t*_*w *_tests. Comparing groups in logistic regression (and any generalized linear model) is equivalent to testing the hypotheses of whether the coefficients *β *= 0. Baggerly *et al. *[15] showed evidence suggesting that "the logit scale may be more appropriate" than the original proportion scale. One drawback with overdispersed logistic regression, however, is that it can break down for cases where all the tag counts in any of groups are very small. In such cases, the deviance test rather than the *t*-test (on the hypothesis that the coefficient *β *is zero) has been proposed [15]. Besides that a systematic evaluation of the deviance test is needed, a potential drawback with the deviance test is that it may require multiple rounds of model fitting if a model contains multiple factors or covariates. Furthermore, questions still remain on exactly when the deviance test should be used in preference to the *t*-test.

In this report we introduce an overdispersed log-linear model approach to analyzing SAGE which is closely related to overdispersed logistic regression but has a different mean-variance relationship assumption. We compare its performance in identifying differential expression with that of three other methods, including the *t*-test, *t*_*w *_test and overdispersed logistic regression. Analysis of simulated and real datasets show that both the log-linear and logistic overdispersion methods generally perform better than the *t *and *t*_*w *_tests. Based on simulated data, the log-linear method is found to have better performance than the logistic method, showing equal or higher statistical power over a range of parameter values and with different data distributions. The overdispersed log-linear method also appears to have better performance on the real SAGE data which we analyze; a number of cases are seen where a tag is identified by the log-linear approach and appears to be clearly differentially expressed, but which would not have been identified as significant using the logistic regression method. Overdispersed log-linear models also offer the same flexibility as logistic regression, allowing for modeling multiple factors and/or covariates. We conclude that the overdispersed log-linear models provide an attractive and reliable framework for analyzing SAGE experiments involving multiple libraries.

## Results

### Overdispersed log-linear models: a case study

Overdispersed log-linear models (see details in Methods) are very similar to overdispersed logistic models, but there are two major differences. First, overdispersed log-linear models work with logarithms of proportions (the log link) with logarithms of sample sizes *m*_*i *_as offsets. In contrast, overdisersped logistic models use the log of the odds (the logit link). Second, the assumption of an overdispersed log-linear model leads to derived weights used by iteratively reweighted least squares (IRLS) that depend on the means of the tag counts (i.e. the weights depend on both library sizes and tag proportions). The weights in overdispersed logistic regression, in contrast, are a function of library sizes only (see Methods).

Baggerly *et al. *[15] illustrated that the overdispersed logistic model can break down in cases where all proportions in one group are 0. Here we show that such a breakdown can also occur when the proportions in one group are small. Table 1 lists the *p*-values obtained from both the deviance and *t *tests. Note that we are testing the hypothesis that *β *= 0. Artificially increasing the tag counts in group 1 so that they approach the level seen in group 2 (which are held fixed), the deviance test in logistic regression and both tests (deviance and *t*) in the log-linear model show the expected trend of an increasing *p*-value (Table 1, columns 5, 6, and 7). In contrast, the *p*-values from the *t*-test in logistic regression actually decrease first and then increase (Table 1, column 4). This discrepancy between results from the *t *and deviance tests in the logistic model (a discrepancy not seen in the log-linear case) suggests that logistic regression can be problematic when the tag counts of all samples in one group are small.

**Table 1:** Comparisons of *t*- and deviance tests in overdispersed logistic regression and log-linear models and a test based on a Bayesian model

	Group 1^a^		logistic regression		log-linear model		Bayesian model
	library 1	library 2	*t*-test^c^	deviance test	*t*-test^c^	deviance test	*E*^d^
				
1^b^	0	0	0.645	0.115	0.003	0.001	0.01
2	2	2	0.485	0.122	0.002	0.002	0.02
3	5	5	0.383	0.133	0.003	0.005	0.04
4	10	10	0.324	0.149	0.007	0.01	0.05
5	20	20	0.291	0.183	0.02	0.025	0.07
6	50	50	0.324	0.29	0.104	0.117	0.11
7	100	100	0.494	0.508	0.376	0.404	0.12

^a^Tag counts in group 1 are artificially increased towards the levels observed in group 2 (which are held fixed). Tag counts in group 2 are 312, 549, 246, 65, 41, and 52. The library sizes and tag counts in group 2 are taken from Baggerly *et al. *[15].

^b ^The empirical tag counts 0.506, and 0.494 are used to replace the zero counts in group 1[15].

^c ^The *t*-test here is testing the hypothesis that *β *= 0.

^d ^*E*, the Bayes Error Rate, is listed. [26].

### Simulation study

To systematically evaluate the performance of the various tests in the case of two-group comparisons, we performed a simulation study. The tests compared here are the *t*, *t*_*w*_, logi*t-t *and log-*t*. For *t *and *t*_*w*_, the test is whether _*p*_*A *= _*p*_*B*, where _*p*_*A *and _*p*_*B *are the mean proportion in groups A and B respectively. The logi*t-t *and log-*t *are *t *tests on the hypothesis of whether *β *= 0 in the overdispersed logistic regression and log-linear models respectively. We do not attempt to replace the *t*-test with the deviance test in the overdispersed logistic regression model since this requires making a possibly subjective decision on when to use one test in preference to the other.

We generated tag counts under three different distributions, choosing different tag proportions and amounts of overdispersion (Table 2). Data generated from the beta-binomial and negative binomial distributions meet the assumptions (i.e. have the mean-variance relationship structure) of the overdispersed logistic regression and log-linear models approaches, respectively. The negative binomial distribution is equivalent to the gamma-Poisson hierarchical model and is considered a robust alternative to the Poisson distribution [16,17]. It should be noted that the *t*_*w*_-test is also derived under the assumption that the data is generated from a beta-binomial distribution [13]. The range of overdispersion parameter values was chosen based on model fits from a real dataset (see section below); we used the 25, 50, and 75 percentile values of the estimated overdispersion  from these fits. Note that the overdispersion parameter *φ *in the logistic model is not directly related to the *φ *in the log-linear model; *φ *values from the two models should not be compared. Given an overdispersion value *φ *and a group mean proportion *p*, the *α *and *β *values for the beta-binomial distribution are derived as *α *= *p*(1/*φ *- 1), and *β *= (1 - *p*)(1/*φ *- 1). The size parameter in the negative binomial distribution is easily derived as 1/*φ*. We used 5 samples (libraries) for each group, and determined the sizes of each of 10 libraries by randomly sampling from a uniform distribution over the interval between 30,000 and 90,000. This yielded library sizes of 66148, 67094, 53338, 80124, 64984, 70452, 74052, 60086, 52966 and 45377; these values were not changed over the course of the simulations. Results (not shown) from a separate run using a different set of library sizes were found to be in agreement with those shown here. A total of 5,000 sets of tag counts were generated for each combination of parameter values. The sensitivity and specificity of each of the tests were then evaluated and compared through receiver operating characteristic (ROC) curves [18].

**Table 2:** A list of parameter values used in the simulations

Distribution	binomial (i.e. no overdispersion); beta-binomial; negative-binomial
overdispersion parameter (*φ*)	8e-06, 2e-05, 4.3e-05 for beta-binomial; 0.17, 0.42, 0.95 for negative binomial
number of samples in groups A and B	5 in each group
mean proportion in group A (*p*_*A*_)	1, 5, 10, 20, 50, and 100 out of 50,000
ratio of mean proportions (*p*_*B *_/ *p*_*A*_)	1, 2 and 4

Note: the library sizes are 66148, 67094, 53338, 80124, 64984, 70452, 74052, 60086, 52966 and 45377, each of which was determined by a draw from a uniform distribution over the interval from 30,000 to 90,000.

**Figure 1:**
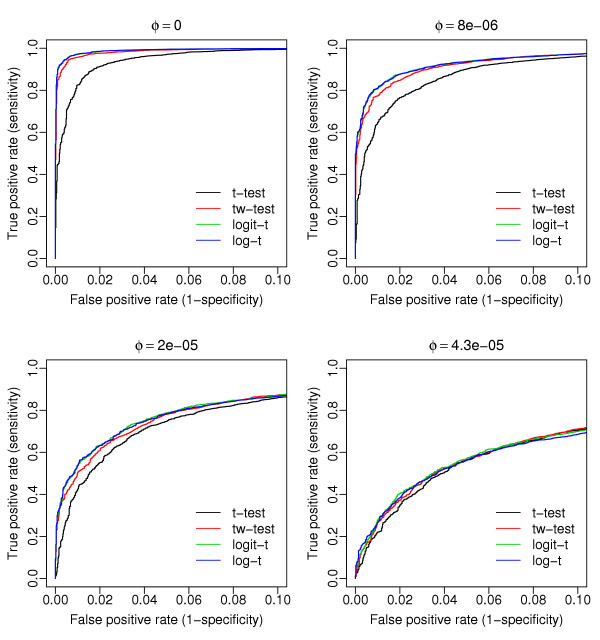
**Comparisons based on simulated data from the beta-binomial distribution**. This figure shows the receiver operating characteristic curves (ROC) of the four tests applied to datasets generated from the beta-binomial distribution with various magnitudes of overdispersion (*φ*) (shown on the top of each graph). For a specific *φ*, 10,000 observations (tags) are simulated; 5,000 are generated under the assumption that *p*_*A *_= *p*_*B *_and the remaining from *p*_*B *_= 2 *p*_*A*_, where *p*_*A *_and *p*_*B *_are the mean proportions of the two groups and *p*_*A *_= 0.0002 (i.e. 10 out of 50,000). For figures generated under other conditions, see Additional file 1.

**Figure 2:**
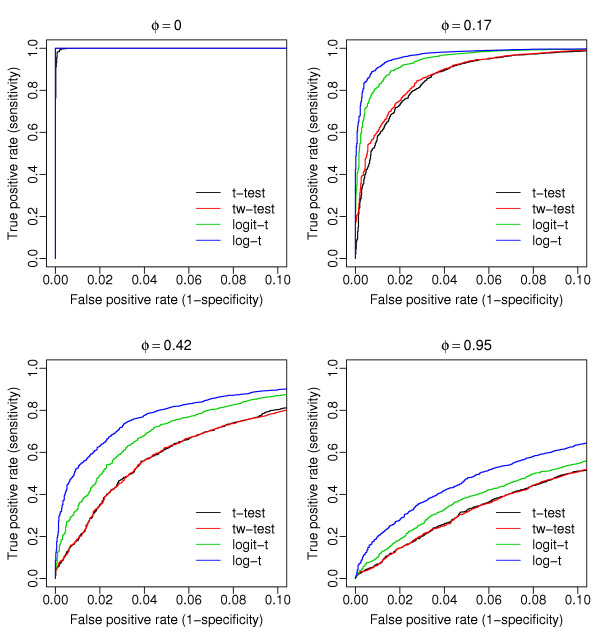
**Comparisons based on simulated data from the negative binomial distribution**. The ROC curves of the four tests are based on datasets generated from the negative binomial distribution with various magnitudes of overdispersion (*φ*). The data are simulated by the same strategy as used in Figure 1, except that *p*_*B *_= 4*p*_*A*_. Note that the overdispersion parameter here is not directly comparable with that in Figure 1 (the parameter *φ *for the negative binomial is not directly related to that for the beta-binomial). For figures generated under other conditions, see Additional file 2.

The ROC curves (one for each of the four tests) shown in Figure 1 were obtained using data generated from the beta-binomial distribution (with overdispersion values *φ *shown on the top of the figure). Given the same false positive rate (*x*-axis), the overdispersion models (logistic and log-linear) clearly show improved statistical power (*y*-axis) compared to the two-sample *t *and *t*_*w *_tests. In contrast, when the four tests are applied to data generated from the negative binomial distribution, the overdispersed log-linear model clearly outperforms the other three tests (Figure 2). Again, the two-sample *t *and *t*_*w *_tests do not perform well in general. The figures generated using other parameter values are available [see Additional files 1 and 2]. These results suggest that for SAGE data, statistics methods based on raw proportions (as in the *t *and *t*_*w *_tests) show less power than the logistic or log-linear model approaches. The overdispersed log-linear model not only shows the best performance in cases where the data are generated in a manner consistent with its assumptions (i.e. from the negative binomial distribution), but also has competitive performance when the data come from a different distribution (here the beta-binomial). This suggests that the overdispersed log-linear model approach is more robust.

### A pancreatic cancer dataset

We further compared the four tests (*t-*test, *t*_*w*_-test, logit-*t*, and log-*t*) using an experimental SAGE data set obtained from the publicly available SAGE Genie website [19]. To identify genes differentially expressed between the pancreatic cancer cells and normal ductal epithelium, Ryu *et al. *[12] compared the gene expression levels of five pancreatic cancer cell lines and two samples of normal pancreatic ductal epithelial cells. The library sizes and numbers of unique tags for the SAGE libraries are shown in Table 3. Note that the numbers in the table are slightly different from those described in the original paper due to the different SAGE tag processing procedures [20]. In this analysis, we ignore tags with total counts less than 3.

**Table 3:** Library information on 5 cancer and 2 normal pancreas SAGE libraries

	Cancer cell lines					Normal cells	
	
Library	ASPC	PL45	CAPAN1	CAPAN2	Panc-1	HX	H126
Library size	31,224	29,557	37,674	23,042	24,749	31,985	32,223
Unique tags	10,622	11,121	14,815	10,157	10,293	12,392	12,360

We first compare the four tests by examining the overlap between the top ranking genes (top 50 and 100) identified by each test (Table 4). For the *t *and *t*_*w *_tests, the genes are ranked by the absolute value of the *t *(or *t*_*w*_) statistics instead of by *p*-values (see Discussion section for details). As shown in Table 4, the results from the logit-*t *and log-*t *tests show the highest agreement (~80%); moderate agreement is observed between *t*_*w *_and logit-*t *or log-*t *tests (~60%) and the least agreement is seen between the *t *and the other three tests (~40%). The top ranking genes identified by the *t*-test are often those with extremely small within-group variances (data not shown). Overall, results from the *t*-test differ the most from the results of the other tests, while the most similar results are seen between the logit-*t *and log-*t *tests. This generally agrees with the trend seen in the simulations.

**Table 4:** Pair-wise comparisons of the four tests

	*t*-test	*t*_*w*_-test	logit-*t*
	
*t*_*w*_-test	39(12)^a^	-	
logit-*t*	42(17)	66(29)	-
log-*t*	36(16)	63(25)	82(43)

^a ^number of genes shared among the list of top 100 and top 50 (in parenthesis) genes identified by the two tests; we note that for the *t *and *t*_*w *_tests, the genes were ranked by the absolute *t *or *t*_*w *_statistic rather than by p-values.

**Figure 3:**
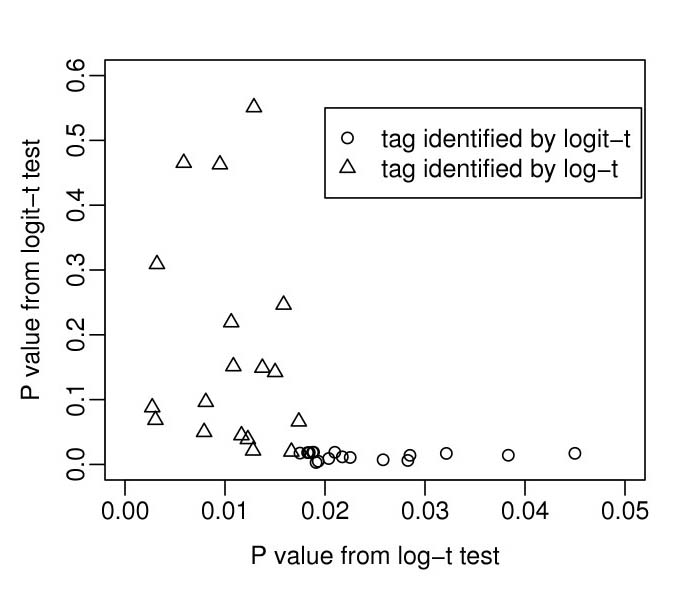
**Comparing *p*-values from the logit-*t *test and those from the log-*t *test**. Of the top 100 tags (ranked according to *p*-values) identified by the logit-*t *test and by the log-*t *test, 82 are common to both leaving 18 tags from each test that are not within the top 100 identified by the other. The *p*-values from both tests for these 36 remaining tags are plotted here. The circles represent the 18 in the top 100 by the logit-*t *test and the triangles those from the log-*t *test. While all the tags identified by the logit-*t *test also have reasonably low p-values according to the log-*t *test, the tags identified by the log-*t *test show a much wider range of *p*-values according to the logit-*t *test.

Of the top 100 genes (ranked by *p*-value) obtained from the logit-*t *and log-*t *tests, 82 genes are in common leaving 18 genes from each test that are not within the top 100 identified by the other test. To further examine the discrepancy between the logit-*t *and log-*t *tests, we plotted *p*-values obtained from both tests for these 36 remaining tags (Fig 3). It can be seen that, while tags identified by the logit-*t *test are also given relatively small *p*-values by the log-*t *test (all less than 0.05), those identified by the log-*t *test show a wide range of *p*-values according to the logit-*t *test. Table 5 lists tags which are ranked among the top 100 by the log-*t *test but which have *p*-values greater than 0.05 by the logit-*t *test; 4 of these were also identified by Ryu *et al. *[12]. Our analysis indicates that the log-*t *test is relatively robust in that it not only gives reasonably small *p*-values to genes identified as significant by the logit-*t *test, but also identifies genes which would never have been considered significant by the logit-*t *test.

**Table 5:** A set of genes identified as significantly differentially expressed (*p *< 0.05 and also among the list of top 100 genes) according to the log-*t *test but not by the logit-*t *test (*p *> 0.05)

			Normal		Cancer				
				
Tag	*P *(log-*t*)	*P *(logit-*t*)	HX	H126	ASPC	PL45	CAPAN1	CAPAN2	Panc-1
AGCAGATCAG*	0.003	0.088	16	9	272	152	138	135	384
TTGGTGAAGG	0.003	0.069	6	0	90	267	194	187	238
CCCATCGTCC	0.003	0.309	13	34	2047	1333	364	456	408
CCTCCAGCTA	0.006	0.465	3	16	452	1766	292	265	364
ACTTTTTCAA	0.008	0.096	25	43	413	379	226	200	65
CAAACCATCC*	0.01	0.463	9	9	439	1235	154	143	133
TGCCCTCAGG	0.011	0.219	16	6	80	196	276	339	4
GCTGTTGCGC*	0.011	0.151	3	3	35	30	82	126	133
GACATCAAGT*	0.013	0.554	0	0	183	548	85	126	20
TTCACTGTGA	0.014	0.149	0	3	128	105	77	91	16
TTGGGGTTTC	0.015	0.142	69	37	701	507	173	195	230
TGCCCTCAAA	0.016	0.246	3	6	32	112	135	178	0
GGGGAAATCG	0.017	0.066	100	71	339	423	119	291	226

Note: Tag counts have been converted to number of tags per 100,000 for the comparison purpose. This scaling is not used in any statistical tests. Tags with (*) are those also identified by Ryu *et al. *[12].

Ryu *et al. *[12] identified 49 up- and 37 down-regulated genes in cancer with the two-sample *t-*test and a set of rule-based methods. We compared their results with those from the log-*t *test (choosing the same number of top genes). Of the total of 86 genes, only 18 are in common (with 9 in each down- and up-regulated gene group). The most significant gene that is up-regulated in cancer on our list (but not in the original paper) is tag, "CTTCCAGCTA", which represents the annexin A2 gene. This gene has been reported to be up-regulated in human pancreatic carcinoma cells and primary pancreatic cancers [21]. Another example is tag 'TTGGTGAAGG', which corresponds to the gene encoding thymosin, beta 4. This gene also has been shown to be "expressed at high levels both in tumor cell lines and in primary cultures of normal pancreas, but not in normal tissue" [22]. A list of the top 20 genes up-regulated and the top 20 genes down-regulated in cancer based on the log-*t *test are listed in Table 6.

**Table 6:** A list of top 40 genes differentially expressed between pancreatic cancer and normal ductal epithelium

Tag	Description	*P*	HX	H126	ASPC	PL45	CAPAN1	CAPAN2	Panc-1
Up-regulated in pancreatic cancer									
CTTCCAGCTA	annexin A2	0.0011	19	25	128	217	143	148	170
AAAAAAAAAA	-	0.0018	6	3	128	210	180	165	133
AGCAGATCAG	S100 calcium binding protein A10 (annexin II ligand, calpactin I, light polypeptide (p11))	0.0027	16	9	272	152	138	135	384
TTGGTGAAGG	thymosin, beta 4, X-linked	0.003	6	0	90	267	194	187	238
CCCATCGTCC	motichondria gene	0.0032	13	34	2047	1333	364	456	408
CCTCCAGCTA	keratin 8	0.0059	3	16	452	1766	292	265	364
GGAAAAAAAA	ATP synthase, H+ transporting, mitochondrial F1 complex, epsilon subunit	0.0063	3	6	64	61	74	74	57
CCCCAGTTGC	calpain, small subunit 1	0.0066	22	22	64	88	77	61	113
AACTAAAAAA	ribosomal protein S27a	0.0078	19	16	45	85	80	61	61
TTCAATAAAA	RPLP1, Ribosomal protein, large, P1	0.0079	9	25	147	179	135	104	40
GCAAAAAAAA	chromosome 21 open reading frame 97	0.0079	6	3	58	68	40	65	65
ACTTTTTCAA	motichondria gene	0.0081	25	43	413	379	226	200	65
CAAACCATCC	KRT18, Keratin 18	0.0095	9	9	439	1235	154	143	133
GTGTGGGGGG	Junction plakoglobin	0.0096	6	3	29	64	50	56	61
TGCCCTCAGG	LCN2, Lipocalin 2 (oncogene 24p3)	0.0106	16	6	80	196	276	339	4
GCTGTTGCGC	-	0.0108	3	3	35	30	82	126	133
AAGAAGATAG	ribosomal protein L23a	0.0116	16	9	77	108	85	65	24
GAAAAAAAAA	SMAD, mothers against DPP homolog 3 (Drosophila)	0.0118	6	0	74	47	40	56	44
ACCTGTATCC	IFITM3, interferon induced transmembrane protein 3 (1-8U)	0.0123	13	3	26	81	64	82	53
CAACTTAGTT	myosin regulatory light chain MRLC2	0.0128	6	6	51	61	53	48	16
									
Down-regulated in pancreatic cancer									
GACGACACGA	ribosomal protein S28	0.0001	428	388	109	122	90	117	154
GGACCACTGA	ribosomal protein L3	0.0002	310	270	102	105	101	104	61
GATCTCTTGG	S100 calcium binding protein A2	0.0002	188	174	3	10	8	4	0
AGCAGGAGCA	S100 calcium binding protein A16	0.0005	144	152	26	41	45	26	16
AGCTGTCCCC	capping protein (actin filament) muscle Z-line, beta	0.0005	219	254	13	3	3	4	0
GACTGCGCGT	tumor necrosis factor receptor superfamily, member 12A	0.0007	103	93	10	10	24	22	16
GTGGTGTGTG	congenital dyserythropoietic anemia, type I	0.0011	59	87	10	10	8	13	8
TAGGCATTCA	-	0.0012	119	115	0	0	0	0	0
TGAGTGGTCA	microtubule-associated protein 1 light chain 3 beta	0.0017	66	53	0	7	5	13	8
GGCGGCTGCA	excision repair cross- complementing rodent repair deficiency, group 1	0.0017	66	53	6	7	3	4	0
AAGTTTGCCT	glutaredoxin (thioltransferase)	0.0022	66	62	0	3	3	0	4
AGCTCTCCCT	Ribosomal protein L17	0.0023	335	357	77	145	82	143	125
CCGAAGTCGA	transcriptional regulating factor 1	0.0024	53	56	0	7	5	0	0
GCTGCTGCGC	-	0.0024	228	320	0	0	0	0	4
TTGGGAGCAG	isoleucine-tRNA synthetase	0.0031	72	43	10	10	19	4	8
TAAGGAGCTG	Ribosomal protein S26	0.0031	344	329	138	85	96	43	101
AACAGAAGCA	hypothetical protein FLJ25692	0.0031	75	59	13	24	24	9	16
CCTCCACCTA	peroxiredoxin 2	0.0031	56	43	16	10	3	9	4
TGTGAGTCAC	-	0.0038	31	62	0	0	0	0	0
TCAGGGATCT	-	0.0038	41	53	0	0	0	0	0

Note: tag counts have been converted to tags per 100,000 for comparison purposes. The *p *values listed are from the log-*t *test.

## Discussion

In this report we introduced a log-linear model with overdispersion for testing differential gene expression in SAGE. This model is closely related to the overdispersed logistic model proposed by Baggerly *et al. *[15] but with a different mean-variance relationship assumption. The differences between two models can be seen clearly in the weight (used by IRLS) associated with each observation: assuming library sizes are reasonably close, the overdispersed log-linear model tends to assign higher weights to observations in the group with the smaller mean proportion; in contrast, approximately equal weights are assigned to all the observations in the overdispersed logistic model. Although for real SAGE data the true mean-variance relationship is unknown, it has been observed that "for the higher counts data, the between-library variability is the dominant part of the variation" [13]; this suggests that the magnitude of the overdispersion in the group with higher counts is probably larger than that in the group with low counts so that the assumptions of the overdispersed log-linear model is probably more appropriate for SAGE data.

**Figure 4:**
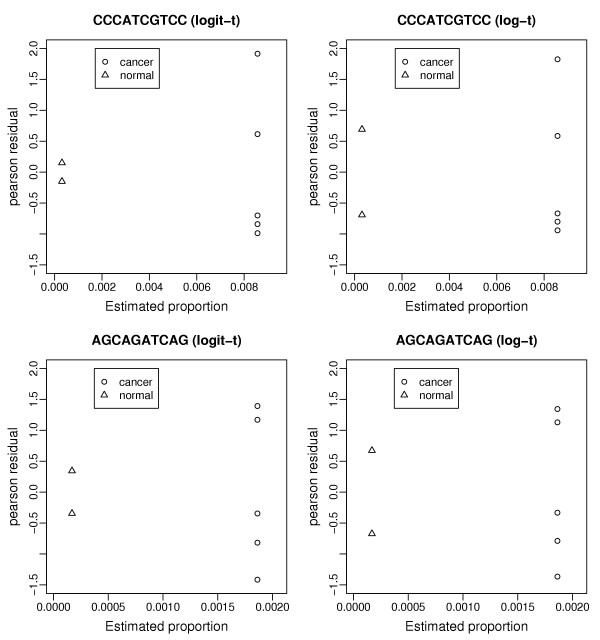
**Plot of standardized residuals against estimated proportions**. Standardized Pearson's residuals (y-axis) plotted vs. the proportion estimates (x-axis) for the two groups. The standardized Pearson's residuals are asymptotically distributed as a standard normal. The model fits of two tags (among the list of genes in Table 5) are shown here; the left is from the fit using the overdispersed logistic model and the right from the overdispersed log-linear model. A lower variance of residuals in the group (normal) with lower mean proportion is an indication of poor model fit.

We also compared the model fits of the overdispersed logistic and log-linear models. Due to the introduction of the overdispersion parameter, the deviance statistic is no longer a valid basis for model fit comparison. An alternative is to use the standardized Pearson residuals, which have an asymptotic standard normal distribution [23]. Williams [24] proposed the approach of plotting the standardized Pearson residuals against the predicted proportions; a problem with a model fit is indicated by a significant decrease in the variance of the standardized residuals as estimated proportions approach zero. Figure 4 shows the residual plots from the logistic and log-linear model fits for two tags (the tag counts are listed in Table 5). In the overdipersed logistic regression case (left panels of Figure 4), the variance of the standardized Pearson's residuals is seen to be much smaller in the normal group than in the cancer group. Such a difference is not evident in the overdispersed log-linear model fits (right panels of Figure 4). Although the sample size is very small in this example (only 2 in the normal group), the residual plots give further indication that log-linear models provide a better fit to SAGE data than logistic models.

From the simulation study we have shown that, besides their limitation to two-group comparison, both the *t*- and *t*_*w*_-tests, in general, are not as powerful as tests which allow for the possibility of overdispersion. We mention one specific problem that can arise with the *t*- and *t*_*w*_-tests if the number of samples in the data set is small. Note that the rank orders from the *t-*test and the *t*_*w *_test in Table 4 are based on test statistics instead of *p*-values. The rank orders based on *p*-values can be different from those based on test statistics if the residual degrees of freedom differ among tests. Both the *t-*test and the *t*_*w*_-test use the Satterthwaite approximation [25] for the number of degrees of freedom since the variances are assumed to be different in the two groups. An example of how this can be problematic is given by tag "AGCTGTCCCC", which has tag counts 70, 82 in the two normal samples, and 4, 1, 1, 1, 0 in the five cancer cell line samples. The differential expression is highly significant based on the logit-*t *(*p*-value 0.0003) and log-*t *(*p*-value 0.0005) tests. In contrast, if the *t*_*w*_-test with the Satterthwaite approximation to the degrees of freedom is used, this tag is barely significant at the 5% level (*p*-value 0.050). The reason is that, while the magnitude of the *t*_*w *_statistic for this tag is actually extremely high (|*t*_*w*_| = 12.01), the calculated degrees of freedom is only about 1 (which leads to low significance). The small value for the degrees of freedom arises here because the estimated variance in the cancer group is very small; the approximated degrees of freedom is then about equal to the sample size of the normal group minus 1 (here, 2-1 = 1). Cases like this occur frequently in this data set since the number of libraries (samples) in one group is very small. It is not uncommon to have small sample numbers with SAGE data.

The four methods compared in this study follow the frequentist approach of hypothesis testing, and can be broadly considered as examples of linear models. For two-group comparisons, Vencio *et al. *[26] introduced a Bayesian approach to rank tags by the Bayes Error Rate. We compared their approach with the methods based on linear models by looking at differences in gene rankings determined using the pancreatic dataset. Considering the top 100 genes identified by the different tests, the two overdispersed models show the best agreement with the Bayesian method (~70% in common); 63 genes (of the top 100) are identified by all three tests. We also evaluated the Bayesian method using the artificial data in Table 1; as the tag counts in group 1 are increased, the evidence for differential expression decreases (i.e. the Bayes Error Rate goes up), which follows the expected trend. Furthermore, if we recognize tags with p < 0.05 or E<0.1 as being significantly differentially expressed [26], the results from the Bayesian approach are more consistent with those from the log-linear model than from the logistic models (see Table 1). Since the evidence measures used are conceptually very different, to perform a direct comparison between "P-value"-based methods and the Bayesian approach is not straightforward. Our results, however, suggest that the Bayesian approach of Vencio is a competitive Bayesian alternative for analyzing SAGE data in the case of two-group comparisons.

**Figure 5:**
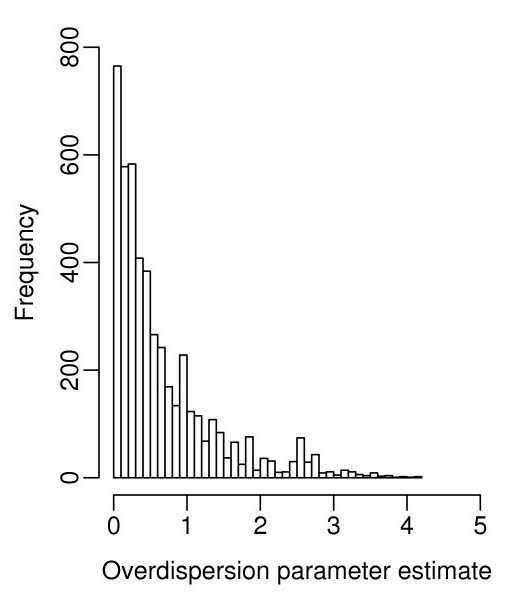
**The distribution of overdispersion estimates **(). The estimates are from the overdispersed log-linear model fit to the pancreas data. Tags with the overdispersion estimate 0 are not shown in the figure.

The current study has not considered the issue of multiple testing problems which is still under active research [27,28]. We note that one possible area for further improvement is to use information across genes (tags) with similar magnitude of dispersion to obtain potentially more robust and accurate overdispersion (and therefore, error) estimates. In all the methods compared here, everything is done one tag at a time, i.e., estimates of the amount of overdispersion are done for each tag individually and these can vary widely (see Figure 5). For expression data with continuous values, strategies on information sharing have been proposed [29-31] and these strategies may be adapted for discrete data such as in SAGE.

## Methods

### Data

Suppose that there are a total of *n *SAGE libraries in an experiment. Let *m*_*i *_be the size (total tag counts) of library *i *(*i *= 1..*n*) and *r*_*i *_be the tag counts for a specific tag in that library.

Also, let ***x*_*i *_**be the associated vector of explanatory variables and *β *the vector of coefficients. The comparison of two groups of SAGE libraries is a special case where there is only one explanatory variable associated with each observation (i.e. one factor with 2 levels).

### The two-sample *t*-test

The *t-*test proposed by Welch [25] was used to test whether the mean of the proportions in one group equals the mean of the other. The proportions are assumed to have unequal variances in the two groups and the degrees of freedom is calculated based on the Satterthwaite approximation as in the *t*_*w*_-test (see below).

### The *t*_*w*_-test

Baggerly *et al. *[13] introduced a beta-binomial sampling model to account for the extra-binomial variation for a simple design in which the comparison is between two groups of SAGE libraries. This is a special case of a linear model that contains one explanatory variable. Briefly, unobserved random variables *P*_*i *_are introduced to account for the between-library variation. For a given group, *P*_*i *_is assumed to have a beta distribution (*α*, *β*) with mean and variance *E*(*P*_*i*_) = *α*/(*α*+*β*), *and Var*(*P*_*i*_) = *αβ */ [(*α*+*β*)^2 ^(*α*+*β*+1)]. Notice that this is a special case of the form *Var(P*_*i*_) = *φ p*_*i*_(1 - *p*_*i*_) as in the overdispersed logistic model, where *φ *= 1/(*α*+*β*+1). Next, the group proportion  is estimated by a weighted linear combination of individual proportions within the group , where  = *r*_*i*_/*m*_*i *_and *w*_*i *_are weights associated with each individual proportion. The unbiased variance estimator of  is given as



To avoid having an estimated variance that is less than the binomial sampling variance, a lower-bound for the variance is also provided. All the parameters (i.e. *α*, *β *and *w*_*i*_) are obtained through an iterative procedure. The same estimation procedure is applied to data from the other group. For testing whether the proportion in one group (say group A) equals the proportion in the other group (group B), a *t-*like statistic *t*_*w *_is constructed, where



The *t*_*w *_statistic is assumed to have a *t-*distribution with the degrees of freedom (*df*) calculated from the Satterthwaite approximation:



where *n*_*A *_and *n*_*B *_are the number of SAGE libraries in the group A and B respectively. This test is called the *t*_*w*_-test here. The implementation of both the *t*- and *t*_*w*_-test can be found in [13].

### Overdispersed logistic regression approach

Baggerly *et al. *[15] provided a thorough description on this approach and details can be found in [24]. Briefly, unobserved continuous random variables *P*_*i *_are introduced to account for the between-library variation, where the mean and variance of *P*_*i *_have the following forms: *E*(*P*_*i*_) = *p*_*i *_; *Var*(*P*_*i*_) = *φ p*_*i*_(1 - *p*_*i*_). Here *φ *is a nonnegative scale parameter. Conditional on *P*_*i*_= *p*_*i*_, the *r*_*i *_have a binomial distribution (*m*_*i*_, *p*_*i*_). The unconditional mean and variance of *r*_*i *_can be shown to be *E*(*r*_*i*_) = *m*_*i *_*p*_*i *_and *Var*(*r*_*i*_) = *m*_*i *_*p*_*i*_(1 - *p*_*i*_) [1+(*m*_*i*_-1) *φ*]. Notice that if *φ *is 0 (i.e. there is no between-library variation or overdispersion), the variance of *r*_*i *_is the usual binomial variance *m*_*i *_*p*_*i*_(1 - *p*_*i*_). The estimation of coefficients *β *is carried out by the iteratively reweighted least-squares (IRLS) procedure, where the weights *w*_*i *_are 1/ [1+(*m*_*i *_- 1) *φ*]. Note that the weights *w*_*i *_are equal if the library sizes *m*_*i *_are equal.

The parameter *φ *is estimated by equating the goodness of fit Pearson's chi-square statistic *X*^2 ^to its approximate expected value, which is



where *v*_*i *_= *m*_*i *_*p*_*i*_(1 - *p*_*i*_), and *d*_*i *_is the variance of the linear predictor . An iterative procedure is introduced to estimate *φ *and *β*, where the estimates of *φ *(and accordingly, the weights *w*_*i*_) and *β *are updated at each step. Given the estimated coefficients, the testing hypothesis is whether one (or more if there are more than two groups) of the coefficients (*β*) is 0. For this, the *t*-test rather than the *z*-test is recommended due to the introduction of the overdispersion parameter into the model [15,32].

The hypothesis test based on overdispersed logistic regression is called the logi*t-t *test here. The implementation including source code can be found in [15]. We consider overdispersion models (logistic or log-linear) only if the Pearson's chi-square statistic from the usual logistic regression (or log-linear) fit (i.e. without overdispersion) is greater than or equal to its expected value, *n-p*.

### Overdispersed log-linear models

This model is closely related to the overdispersed logistic regression model. One way to derive it is based on the gamma-Poisson hierarchical model assumption [16]. Assume that an unobserved random variables *θ*_*i *_is distributed according to

*θ*_*i *_~ Gamma(*μ*_*i*_, 1/*φ*),

where *μ*_*i *_= *m*_*i *_*p*_*i*_, *φ *>0, *E*(*θ*_*i*_) = *μ*_*i *_and *Var*(*θ*_*i*_) = . Given *p*_*i*_, the response variable *r*_*i *_is assumed to be distributed as

*r*_*i *_| *p*_*i *_~ Poisson(*μ*_*i*_).

The unconditional mean and variance of *r*_*i *_can be shown to be *E*(*r*_*i*_) = *μ*_*i *_= *m*_*i *_*p*_*i *_and *Var*(*r*_*i*_) = *μ*_*i *_(1+*μ*_*i*_*φ*). Notice that as *φ *decreases to 0, the variance of *r*_*i *_approaches the usual Poisson variance *μ*_*i *_(i.e. *m*_*i *_*p*_*i*_). The same mean-variance relationship can also be derived if we assume *r*_*i *_has a negative- binomial distribution [16]. The mean *μ*_*i *_of the response variable *r*_*i *_and the covariates ***x***_*i *_are connected through the log link function,

log *μ*_*i *_= log(*m*_*i *_*p*_*i*_) = *x*_*i*_*β*.

As in the overdispersed logistic regression model, the estimates of the coefficients *β *are obtained by the iteratively reweighted least-squares procedure, where the weights *w*_*i *_are 1/(1+*μ*_*i *_*φ*) [33]. Note that, in contrast to the overdispersed logistic regression model where the weights only depend on library sizes *m*_*i*_, the weights in the log-linear model depend on *μ*_*i *_(i.e. both *m*_*i *_and *p*_*i*_).

The hypothesis test based on overdispersed log-linear models is called the log-*t *test here. The R [34] source code and a web-interface for implementing this approach are available [35].

## Authors' contributions

JL developed the method. JL and JKT carried out the simulation and data analysis. JKT and JL set up the web interface for implementing this approach. TBK supervised the study, and assisted with the methodology. All authors contributed to the writing, read and approved the final manuscript.

## Supplementary Material

Additional File 1 This gzipped tar file contains figures showing the receiver operating characteristic curves (ROC) for the four tests applied to datasets generated from the beta-binomial distribution with various magnitudes of overdispersion(*φ*) and mean proportions. For example, the file 2_8e-06_0.0002.png shows the ROC curves when *p*_*B *_= 2*p*_*A*_, *φ *= 8e-06 and *p*_*A *_= 0.0002. Click here for file

Additional File 2 Similar to the file above, this file contains figures of ROC curves but with data generated from the negative binomial distribution. Click here for file
