# Supplementary figures and images for: Identifying differential expression in multiple SAGE libraries: an overdispersed log-linear model approach
Source: BMC Bioinformatics. 2005 Jun 29;6:165. doi: 10.1186/1471-2105-6-165 (PMC1189357; doi:10.1186/1471-2105-6-165)

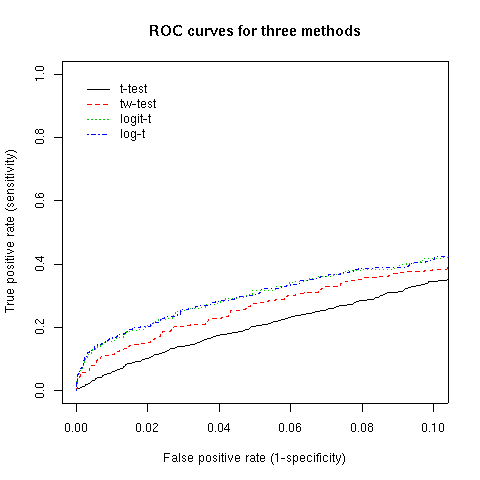

Supplement: Additional File 1 — This gzipped tar file contains figures showing the receiver operating characteristic curves (ROC) for the four tests applied to datasets generated from the beta-binomial distribution with various magnitudes of overdispersion(φ) and mean proportions. For example, the file 2_8e-06_0.0002.png shows the ROC curves when pB = 2pA, φ = 8e-06 and pA = 0.0002. [file 1471-2105-6-165-S1.gz › bin_fig/2_0_2e-05.png]

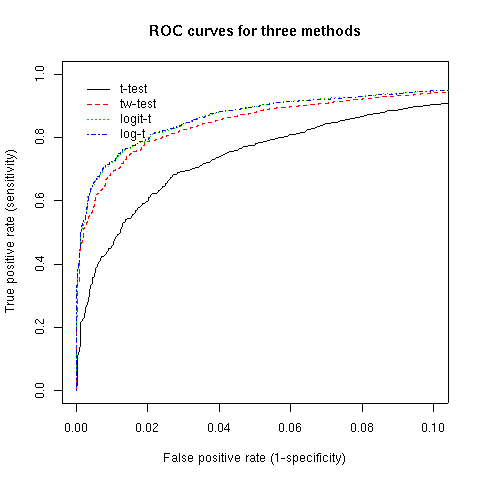

Supplement: Additional File 1 — This gzipped tar file contains figures showing the receiver operating characteristic curves (ROC) for the four tests applied to datasets generated from the beta-binomial distribution with various magnitudes of overdispersion(φ) and mean proportions. For example, the file 2_8e-06_0.0002.png shows the ROC curves when pB = 2pA, φ = 8e-06 and pA = 0.0002. [file 1471-2105-6-165-S1.gz › bin_fig/2_0_0.0001.png]

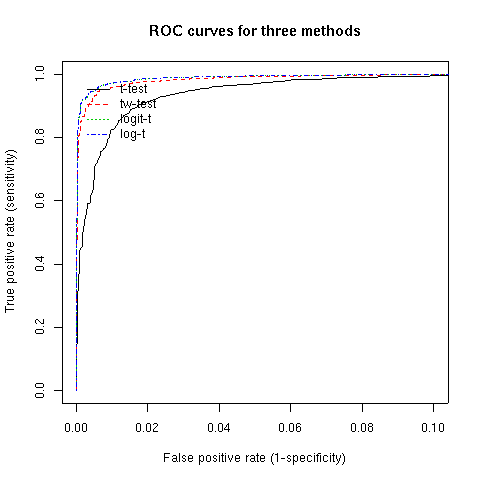

Supplement: Additional File 1 — This gzipped tar file contains figures showing the receiver operating characteristic curves (ROC) for the four tests applied to datasets generated from the beta-binomial distribution with various magnitudes of overdispersion(φ) and mean proportions. For example, the file 2_8e-06_0.0002.png shows the ROC curves when pB = 2pA, φ = 8e-06 and pA = 0.0002. [file 1471-2105-6-165-S1.gz › bin_fig/2_0_0.0002.png]

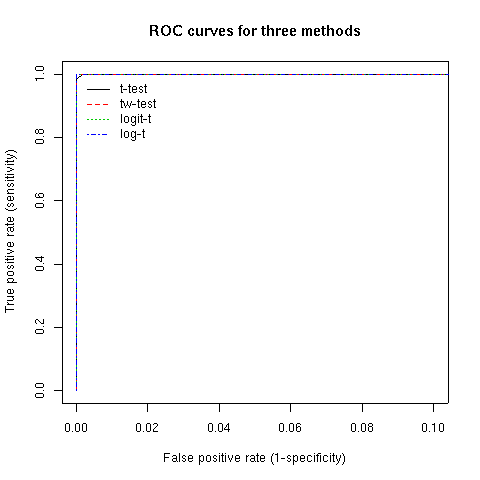

Supplement: Additional File 1 — This gzipped tar file contains figures showing the receiver operating characteristic curves (ROC) for the four tests applied to datasets generated from the beta-binomial distribution with various magnitudes of overdispersion(φ) and mean proportions. For example, the file 2_8e-06_0.0002.png shows the ROC curves when pB = 2pA, φ = 8e-06 and pA = 0.0002. [file 1471-2105-6-165-S1.gz › bin_fig/2_0_0.001.png]

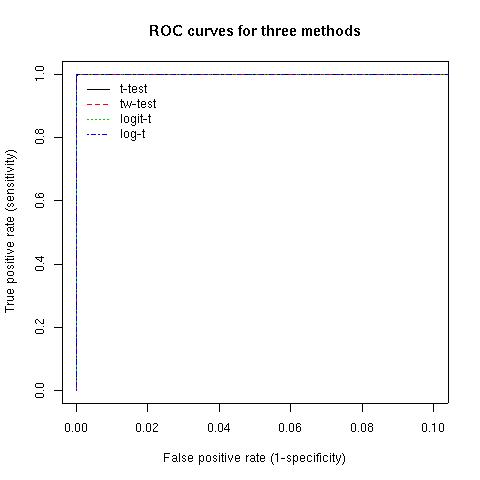

Supplement: Additional File 1 — This gzipped tar file contains figures showing the receiver operating characteristic curves (ROC) for the four tests applied to datasets generated from the beta-binomial distribution with various magnitudes of overdispersion(φ) and mean proportions. For example, the file 2_8e-06_0.0002.png shows the ROC curves when pB = 2pA, φ = 8e-06 and pA = 0.0002. [file 1471-2105-6-165-S1.gz › bin_fig/2_0_0.002.png]

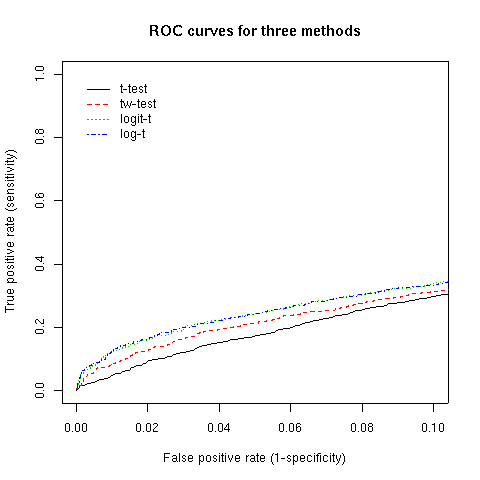

Supplement: Additional File 1 — This gzipped tar file contains figures showing the receiver operating characteristic curves (ROC) for the four tests applied to datasets generated from the beta-binomial distribution with various magnitudes of overdispersion(φ) and mean proportions. For example, the file 2_8e-06_0.0002.png shows the ROC curves when pB = 2pA, φ = 8e-06 and pA = 0.0002. [file 1471-2105-6-165-S1.gz › bin_fig/2_8e-06_2e-05.png]

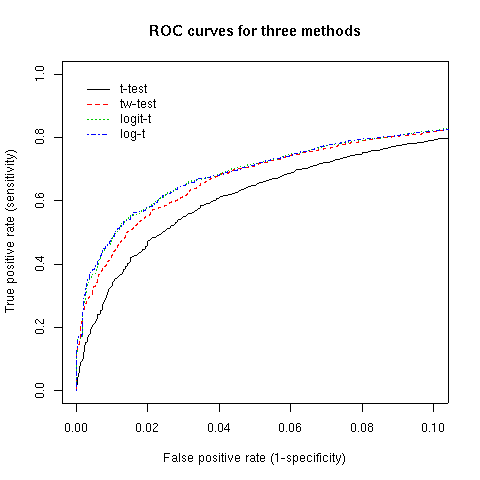

Supplement: Additional File 1 — This gzipped tar file contains figures showing the receiver operating characteristic curves (ROC) for the four tests applied to datasets generated from the beta-binomial distribution with various magnitudes of overdispersion(φ) and mean proportions. For example, the file 2_8e-06_0.0002.png shows the ROC curves when pB = 2pA, φ = 8e-06 and pA = 0.0002. [file 1471-2105-6-165-S1.gz › bin_fig/2_8e-06_0.0001.png]

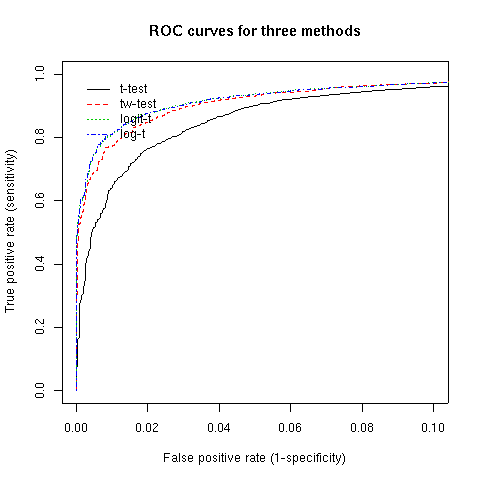

Supplement: Additional File 1 — This gzipped tar file contains figures showing the receiver operating characteristic curves (ROC) for the four tests applied to datasets generated from the beta-binomial distribution with various magnitudes of overdispersion(φ) and mean proportions. For example, the file 2_8e-06_0.0002.png shows the ROC curves when pB = 2pA, φ = 8e-06 and pA = 0.0002. [file 1471-2105-6-165-S1.gz › bin_fig/2_8e-06_0.0002.png]

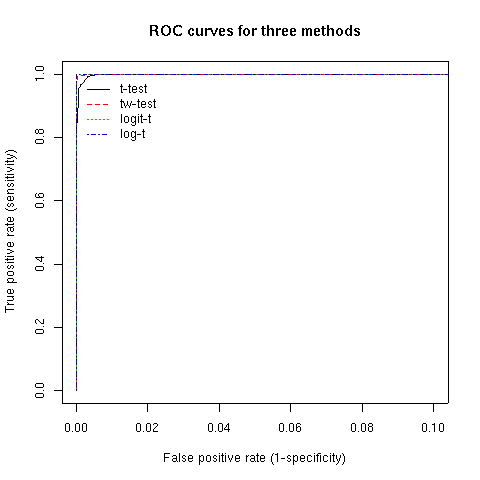

Supplement: Additional File 1 — This gzipped tar file contains figures showing the receiver operating characteristic curves (ROC) for the four tests applied to datasets generated from the beta-binomial distribution with various magnitudes of overdispersion(φ) and mean proportions. For example, the file 2_8e-06_0.0002.png shows the ROC curves when pB = 2pA, φ = 8e-06 and pA = 0.0002. [file 1471-2105-6-165-S1.gz › bin_fig/2_8e-06_0.001.png]

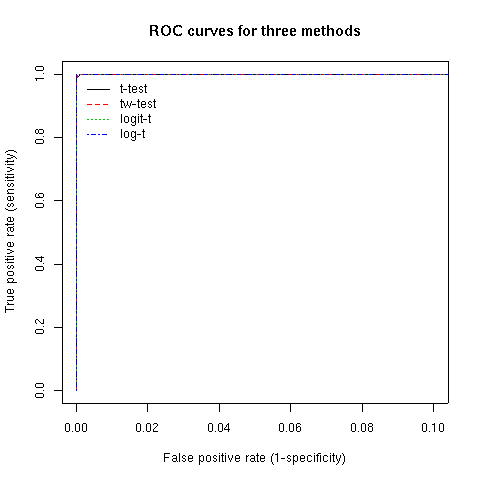

Supplement: Additional File 1 — This gzipped tar file contains figures showing the receiver operating characteristic curves (ROC) for the four tests applied to datasets generated from the beta-binomial distribution with various magnitudes of overdispersion(φ) and mean proportions. For example, the file 2_8e-06_0.0002.png shows the ROC curves when pB = 2pA, φ = 8e-06 and pA = 0.0002. [file 1471-2105-6-165-S1.gz › bin_fig/2_8e-06_0.002.png]

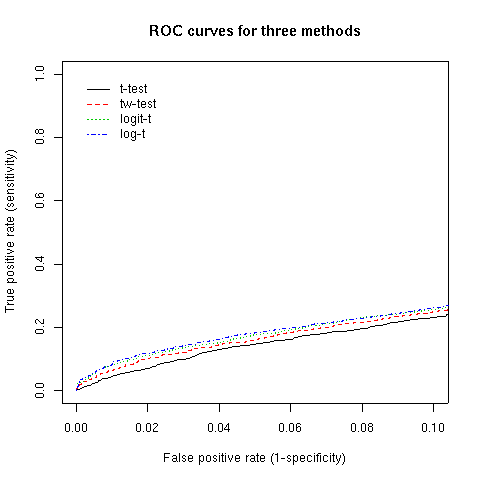

Supplement: Additional File 1 — This gzipped tar file contains figures showing the receiver operating characteristic curves (ROC) for the four tests applied to datasets generated from the beta-binomial distribution with various magnitudes of overdispersion(φ) and mean proportions. For example, the file 2_8e-06_0.0002.png shows the ROC curves when pB = 2pA, φ = 8e-06 and pA = 0.0002. [file 1471-2105-6-165-S1.gz › bin_fig/2_2e-05_2e-05.png]

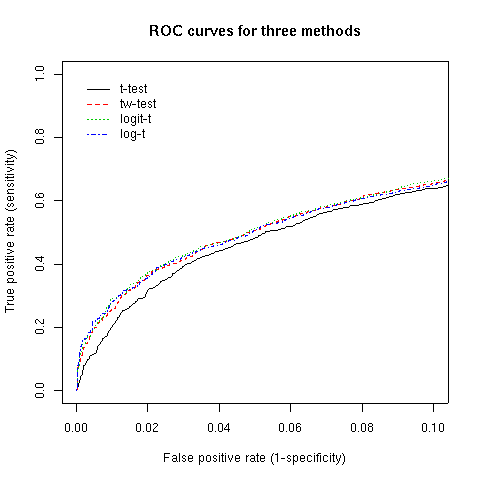

Supplement: Additional File 1 — This gzipped tar file contains figures showing the receiver operating characteristic curves (ROC) for the four tests applied to datasets generated from the beta-binomial distribution with various magnitudes of overdispersion(φ) and mean proportions. For example, the file 2_8e-06_0.0002.png shows the ROC curves when pB = 2pA, φ = 8e-06 and pA = 0.0002. [file 1471-2105-6-165-S1.gz › bin_fig/2_2e-05_0.0001.png]

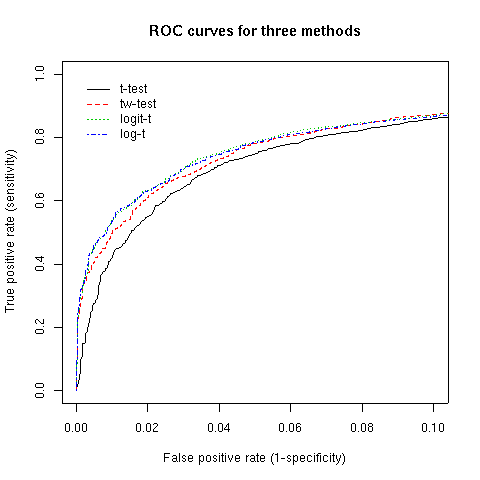

Supplement: Additional File 1 — This gzipped tar file contains figures showing the receiver operating characteristic curves (ROC) for the four tests applied to datasets generated from the beta-binomial distribution with various magnitudes of overdispersion(φ) and mean proportions. For example, the file 2_8e-06_0.0002.png shows the ROC curves when pB = 2pA, φ = 8e-06 and pA = 0.0002. [file 1471-2105-6-165-S1.gz › bin_fig/2_2e-05_0.0002.png]

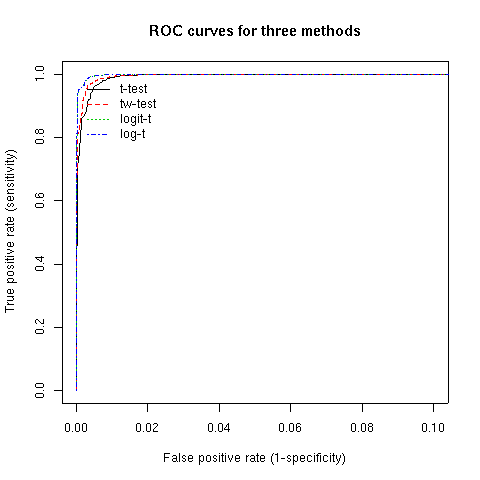

Supplement: Additional File 1 — This gzipped tar file contains figures showing the receiver operating characteristic curves (ROC) for the four tests applied to datasets generated from the beta-binomial distribution with various magnitudes of overdispersion(φ) and mean proportions. For example, the file 2_8e-06_0.0002.png shows the ROC curves when pB = 2pA, φ = 8e-06 and pA = 0.0002. [file 1471-2105-6-165-S1.gz › bin_fig/2_2e-05_0.001.png]

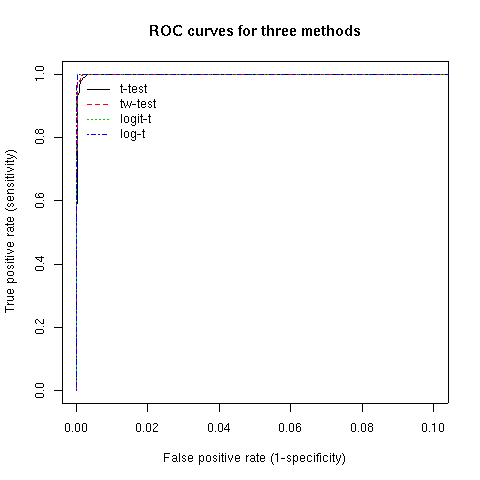

Supplement: Additional File 1 — This gzipped tar file contains figures showing the receiver operating characteristic curves (ROC) for the four tests applied to datasets generated from the beta-binomial distribution with various magnitudes of overdispersion(φ) and mean proportions. For example, the file 2_8e-06_0.0002.png shows the ROC curves when pB = 2pA, φ = 8e-06 and pA = 0.0002. [file 1471-2105-6-165-S1.gz › bin_fig/2_2e-05_0.002.png]

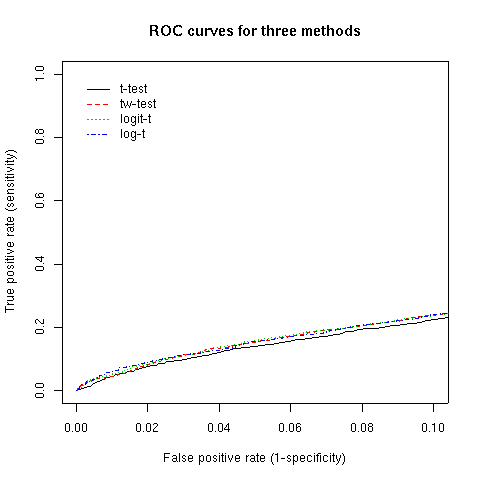

Supplement: Additional File 1 — This gzipped tar file contains figures showing the receiver operating characteristic curves (ROC) for the four tests applied to datasets generated from the beta-binomial distribution with various magnitudes of overdispersion(φ) and mean proportions. For example, the file 2_8e-06_0.0002.png shows the ROC curves when pB = 2pA, φ = 8e-06 and pA = 0.0002. [file 1471-2105-6-165-S1.gz › bin_fig/2_4.3e-05_2e-05.png]

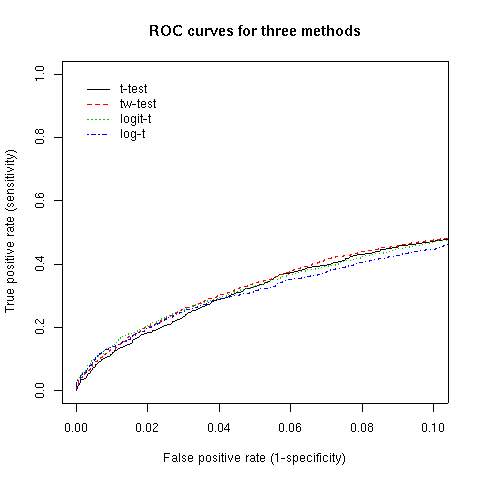

Supplement: Additional File 1 — This gzipped tar file contains figures showing the receiver operating characteristic curves (ROC) for the four tests applied to datasets generated from the beta-binomial distribution with various magnitudes of overdispersion(φ) and mean proportions. For example, the file 2_8e-06_0.0002.png shows the ROC curves when pB = 2pA, φ = 8e-06 and pA = 0.0002. [file 1471-2105-6-165-S1.gz › bin_fig/2_4.3e-05_0.0001.png]

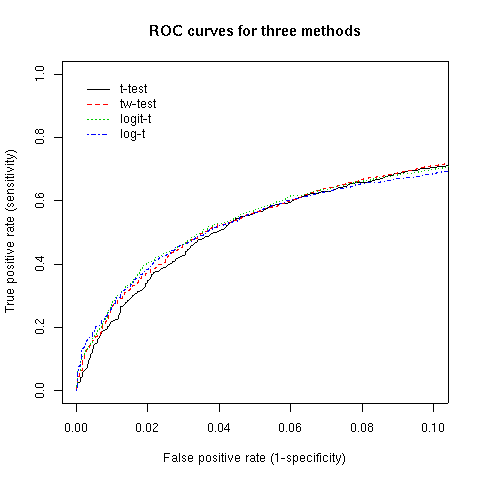

Supplement: Additional File 1 — This gzipped tar file contains figures showing the receiver operating characteristic curves (ROC) for the four tests applied to datasets generated from the beta-binomial distribution with various magnitudes of overdispersion(φ) and mean proportions. For example, the file 2_8e-06_0.0002.png shows the ROC curves when pB = 2pA, φ = 8e-06 and pA = 0.0002. [file 1471-2105-6-165-S1.gz › bin_fig/2_4.3e-05_0.0002.png]

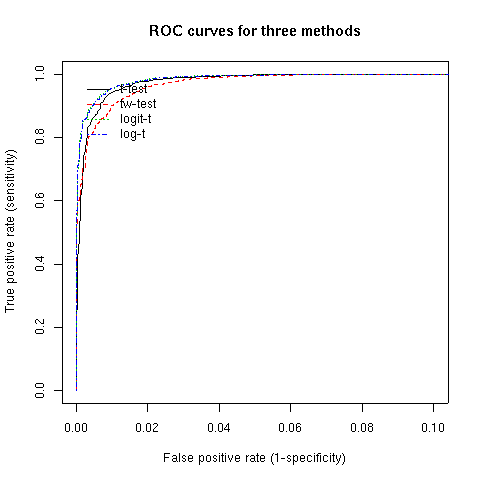

Supplement: Additional File 1 — This gzipped tar file contains figures showing the receiver operating characteristic curves (ROC) for the four tests applied to datasets generated from the beta-binomial distribution with various magnitudes of overdispersion(φ) and mean proportions. For example, the file 2_8e-06_0.0002.png shows the ROC curves when pB = 2pA, φ = 8e-06 and pA = 0.0002. [file 1471-2105-6-165-S1.gz › bin_fig/2_4.3e-05_0.001.png]

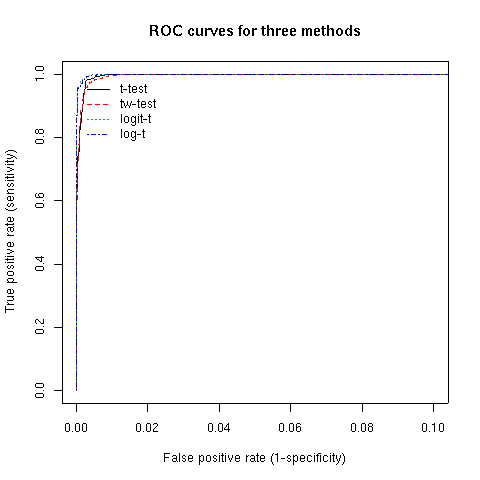

Supplement: Additional File 1 — This gzipped tar file contains figures showing the receiver operating characteristic curves (ROC) for the four tests applied to datasets generated from the beta-binomial distribution with various magnitudes of overdispersion(φ) and mean proportions. For example, the file 2_8e-06_0.0002.png shows the ROC curves when pB = 2pA, φ = 8e-06 and pA = 0.0002. [file 1471-2105-6-165-S1.gz › bin_fig/2_4.3e-05_0.002.png]

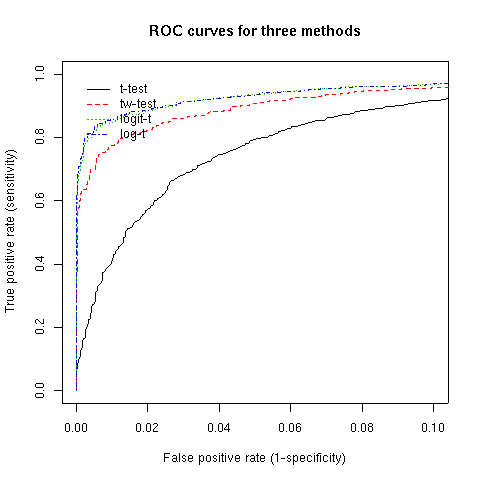

Supplement: Additional File 1 — This gzipped tar file contains figures showing the receiver operating characteristic curves (ROC) for the four tests applied to datasets generated from the beta-binomial distribution with various magnitudes of overdispersion(φ) and mean proportions. For example, the file 2_8e-06_0.0002.png shows the ROC curves when pB = 2pA, φ = 8e-06 and pA = 0.0002. [file 1471-2105-6-165-S1.gz › bin_fig/4_0_2e-05.png]

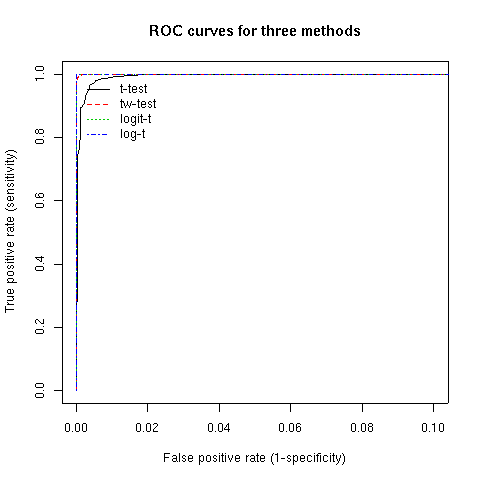

Supplement: Additional File 1 — This gzipped tar file contains figures showing the receiver operating characteristic curves (ROC) for the four tests applied to datasets generated from the beta-binomial distribution with various magnitudes of overdispersion(φ) and mean proportions. For example, the file 2_8e-06_0.0002.png shows the ROC curves when pB = 2pA, φ = 8e-06 and pA = 0.0002. [file 1471-2105-6-165-S1.gz › bin_fig/4_0_0.0001.png]

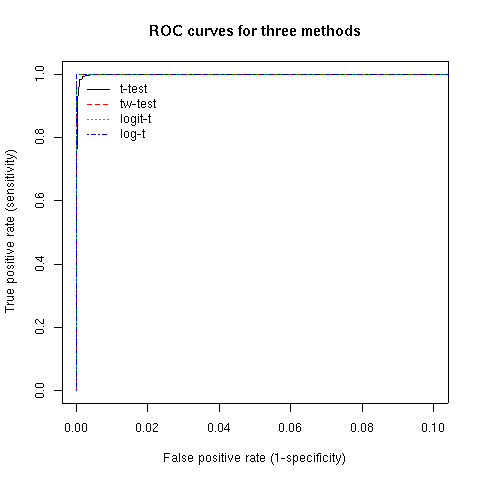

Supplement: Additional File 1 — This gzipped tar file contains figures showing the receiver operating characteristic curves (ROC) for the four tests applied to datasets generated from the beta-binomial distribution with various magnitudes of overdispersion(φ) and mean proportions. For example, the file 2_8e-06_0.0002.png shows the ROC curves when pB = 2pA, φ = 8e-06 and pA = 0.0002. [file 1471-2105-6-165-S1.gz › bin_fig/4_0_0.0002.png]

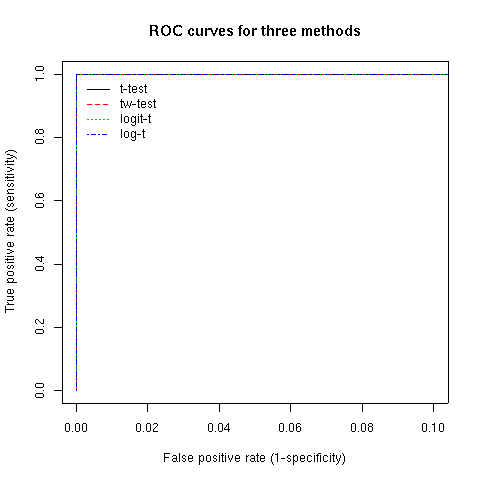

Supplement: Additional File 1 — This gzipped tar file contains figures showing the receiver operating characteristic curves (ROC) for the four tests applied to datasets generated from the beta-binomial distribution with various magnitudes of overdispersion(φ) and mean proportions. For example, the file 2_8e-06_0.0002.png shows the ROC curves when pB = 2pA, φ = 8e-06 and pA = 0.0002. [file 1471-2105-6-165-S1.gz › bin_fig/4_0_0.001.png]

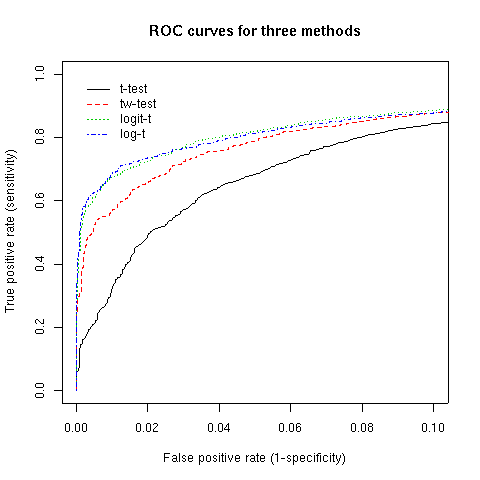

Supplement: Additional File 1 — This gzipped tar file contains figures showing the receiver operating characteristic curves (ROC) for the four tests applied to datasets generated from the beta-binomial distribution with various magnitudes of overdispersion(φ) and mean proportions. For example, the file 2_8e-06_0.0002.png shows the ROC curves when pB = 2pA, φ = 8e-06 and pA = 0.0002. [file 1471-2105-6-165-S1.gz › bin_fig/4_8e-06_2e-05.png]

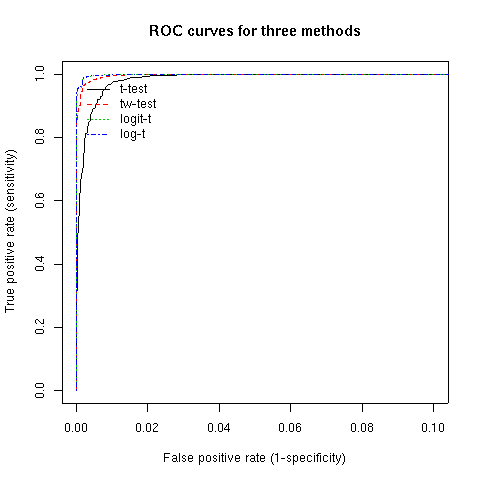

Supplement: Additional File 1 — This gzipped tar file contains figures showing the receiver operating characteristic curves (ROC) for the four tests applied to datasets generated from the beta-binomial distribution with various magnitudes of overdispersion(φ) and mean proportions. For example, the file 2_8e-06_0.0002.png shows the ROC curves when pB = 2pA, φ = 8e-06 and pA = 0.0002. [file 1471-2105-6-165-S1.gz › bin_fig/4_8e-06_0.0001.png]

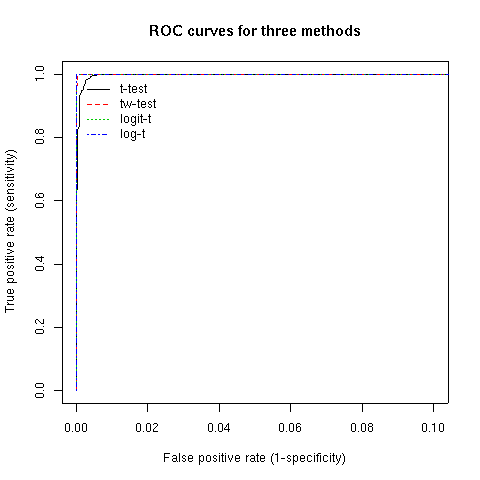

Supplement: Additional File 1 — This gzipped tar file contains figures showing the receiver operating characteristic curves (ROC) for the four tests applied to datasets generated from the beta-binomial distribution with various magnitudes of overdispersion(φ) and mean proportions. For example, the file 2_8e-06_0.0002.png shows the ROC curves when pB = 2pA, φ = 8e-06 and pA = 0.0002. [file 1471-2105-6-165-S1.gz › bin_fig/4_8e-06_0.0002.png]

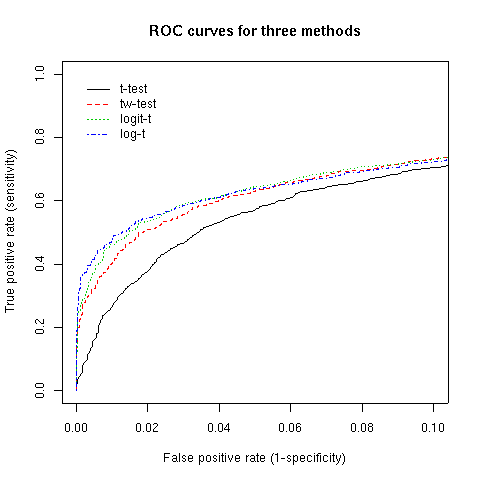

Supplement: Additional File 1 — This gzipped tar file contains figures showing the receiver operating characteristic curves (ROC) for the four tests applied to datasets generated from the beta-binomial distribution with various magnitudes of overdispersion(φ) and mean proportions. For example, the file 2_8e-06_0.0002.png shows the ROC curves when pB = 2pA, φ = 8e-06 and pA = 0.0002. [file 1471-2105-6-165-S1.gz › bin_fig/4_2e-05_2e-05.png]

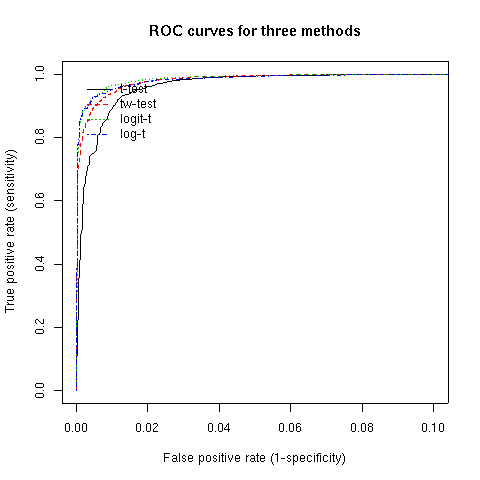

Supplement: Additional File 1 — This gzipped tar file contains figures showing the receiver operating characteristic curves (ROC) for the four tests applied to datasets generated from the beta-binomial distribution with various magnitudes of overdispersion(φ) and mean proportions. For example, the file 2_8e-06_0.0002.png shows the ROC curves when pB = 2pA, φ = 8e-06 and pA = 0.0002. [file 1471-2105-6-165-S1.gz › bin_fig/4_2e-05_0.0001.png]

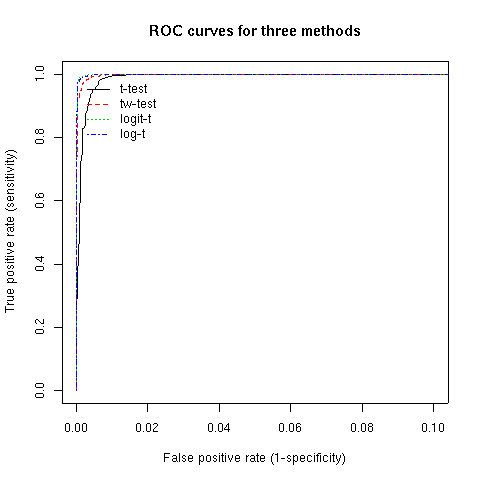

Supplement: Additional File 1 — This gzipped tar file contains figures showing the receiver operating characteristic curves (ROC) for the four tests applied to datasets generated from the beta-binomial distribution with various magnitudes of overdispersion(φ) and mean proportions. For example, the file 2_8e-06_0.0002.png shows the ROC curves when pB = 2pA, φ = 8e-06 and pA = 0.0002. [file 1471-2105-6-165-S1.gz › bin_fig/4_2e-05_0.0002.png]

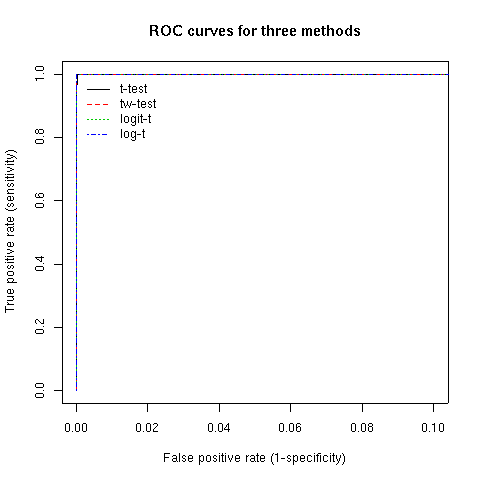

Supplement: Additional File 1 — This gzipped tar file contains figures showing the receiver operating characteristic curves (ROC) for the four tests applied to datasets generated from the beta-binomial distribution with various magnitudes of overdispersion(φ) and mean proportions. For example, the file 2_8e-06_0.0002.png shows the ROC curves when pB = 2pA, φ = 8e-06 and pA = 0.0002. [file 1471-2105-6-165-S1.gz › bin_fig/4_2e-05_0.001.png]

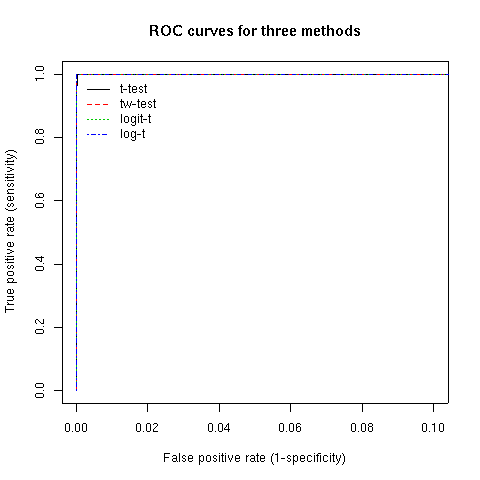

Supplement: Additional File 1 — This gzipped tar file contains figures showing the receiver operating characteristic curves (ROC) for the four tests applied to datasets generated from the beta-binomial distribution with various magnitudes of overdispersion(φ) and mean proportions. For example, the file 2_8e-06_0.0002.png shows the ROC curves when pB = 2pA, φ = 8e-06 and pA = 0.0002. [file 1471-2105-6-165-S1.gz › bin_fig/4_2e-05_0.002.png]

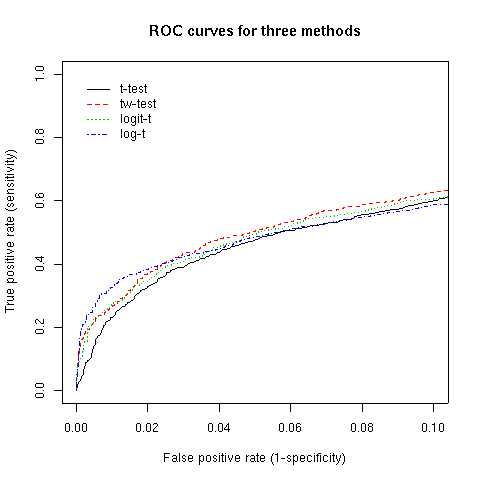

Supplement: Additional File 1 — This gzipped tar file contains figures showing the receiver operating characteristic curves (ROC) for the four tests applied to datasets generated from the beta-binomial distribution with various magnitudes of overdispersion(φ) and mean proportions. For example, the file 2_8e-06_0.0002.png shows the ROC curves when pB = 2pA, φ = 8e-06 and pA = 0.0002. [file 1471-2105-6-165-S1.gz › bin_fig/4_4.3e-05_2e-05.png]

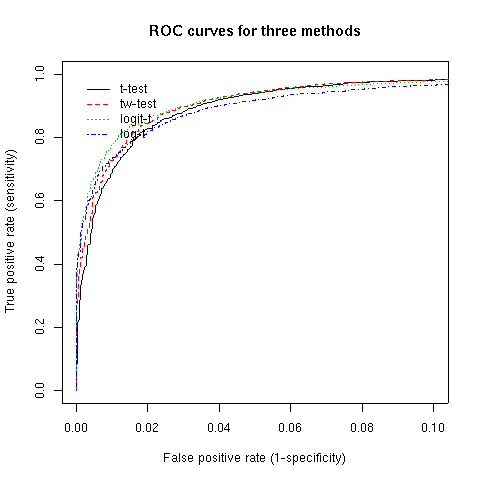

Supplement: Additional File 1 — This gzipped tar file contains figures showing the receiver operating characteristic curves (ROC) for the four tests applied to datasets generated from the beta-binomial distribution with various magnitudes of overdispersion(φ) and mean proportions. For example, the file 2_8e-06_0.0002.png shows the ROC curves when pB = 2pA, φ = 8e-06 and pA = 0.0002. [file 1471-2105-6-165-S1.gz › bin_fig/4_4.3e-05_0.0001.png]

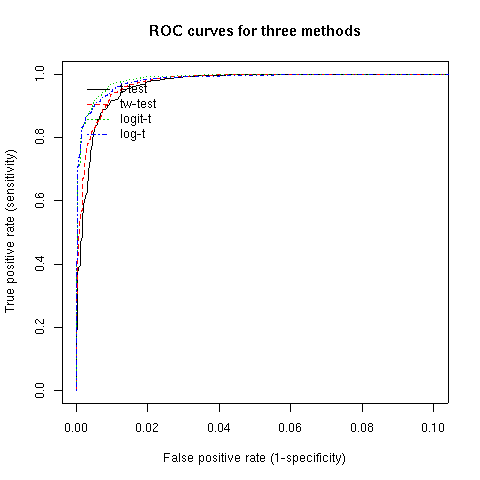

Supplement: Additional File 1 — This gzipped tar file contains figures showing the receiver operating characteristic curves (ROC) for the four tests applied to datasets generated from the beta-binomial distribution with various magnitudes of overdispersion(φ) and mean proportions. For example, the file 2_8e-06_0.0002.png shows the ROC curves when pB = 2pA, φ = 8e-06 and pA = 0.0002. [file 1471-2105-6-165-S1.gz › bin_fig/4_4.3e-05_0.0002.png]

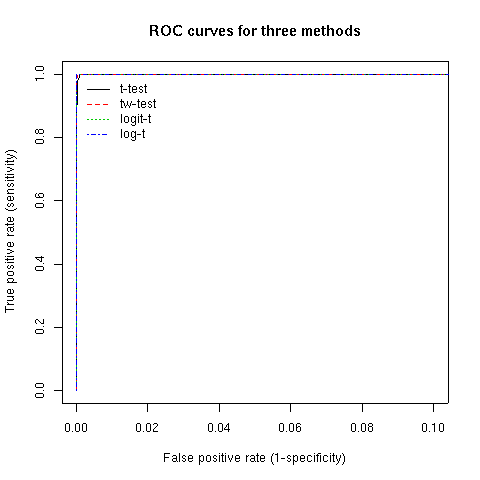

Supplement: Additional File 1 — This gzipped tar file contains figures showing the receiver operating characteristic curves (ROC) for the four tests applied to datasets generated from the beta-binomial distribution with various magnitudes of overdispersion(φ) and mean proportions. For example, the file 2_8e-06_0.0002.png shows the ROC curves when pB = 2pA, φ = 8e-06 and pA = 0.0002. [file 1471-2105-6-165-S1.gz › bin_fig/4_4.3e-05_0.001.png]

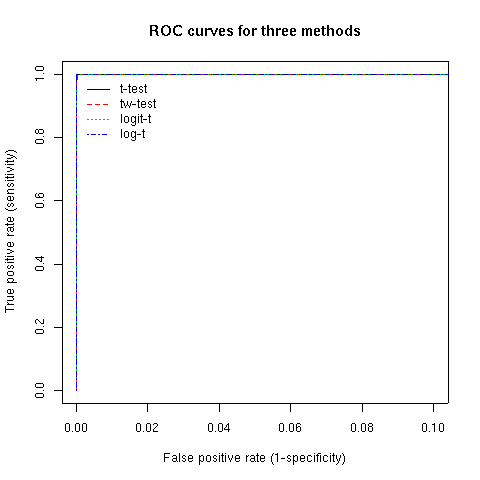

Supplement: Additional File 1 — This gzipped tar file contains figures showing the receiver operating characteristic curves (ROC) for the four tests applied to datasets generated from the beta-binomial distribution with various magnitudes of overdispersion(φ) and mean proportions. For example, the file 2_8e-06_0.0002.png shows the ROC curves when pB = 2pA, φ = 8e-06 and pA = 0.0002. [file 1471-2105-6-165-S1.gz › bin_fig/4_4.3e-05_0.002.png]

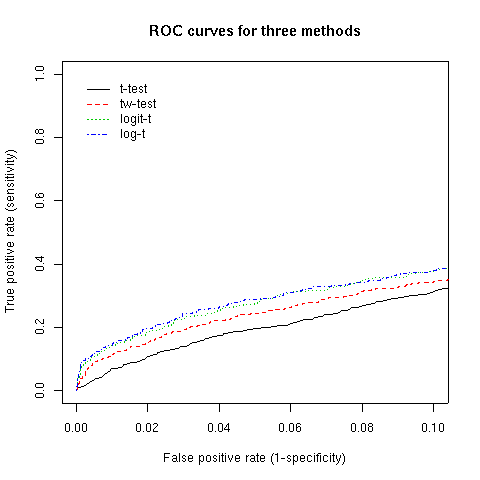

Supplement: Additional File 2 — Similar to the file above, this file contains figures of ROC curves but with data generated from the negative binomial distribution. [file 1471-2105-6-165-S2.gz › ps_fig/2_0.17_2e-05.png]

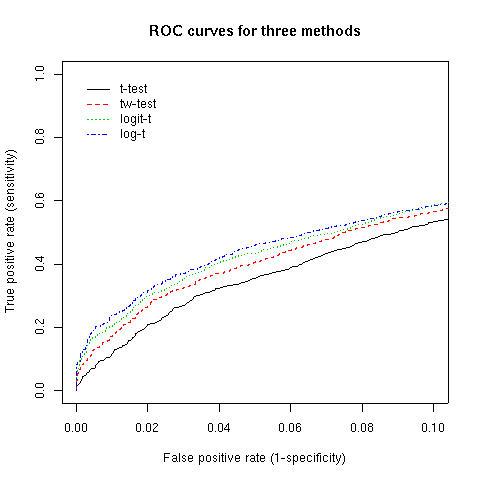

Supplement: Additional File 2 — Similar to the file above, this file contains figures of ROC curves but with data generated from the negative binomial distribution. [file 1471-2105-6-165-S2.gz › ps_fig/2_0.17_0.0001.png]

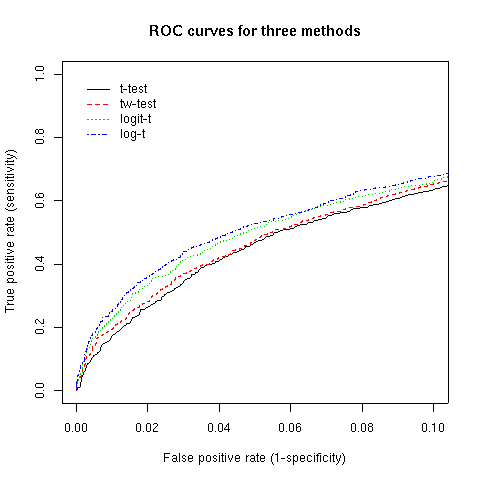

Supplement: Additional File 2 — Similar to the file above, this file contains figures of ROC curves but with data generated from the negative binomial distribution. [file 1471-2105-6-165-S2.gz › ps_fig/2_0.17_0.0002.png]

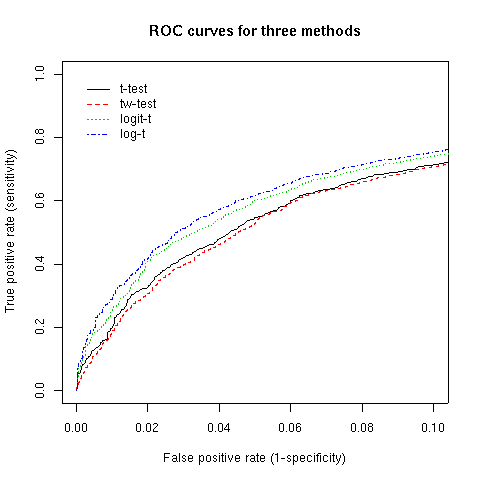

Supplement: Additional File 2 — Similar to the file above, this file contains figures of ROC curves but with data generated from the negative binomial distribution. [file 1471-2105-6-165-S2.gz › ps_fig/2_0.17_0.001.png]

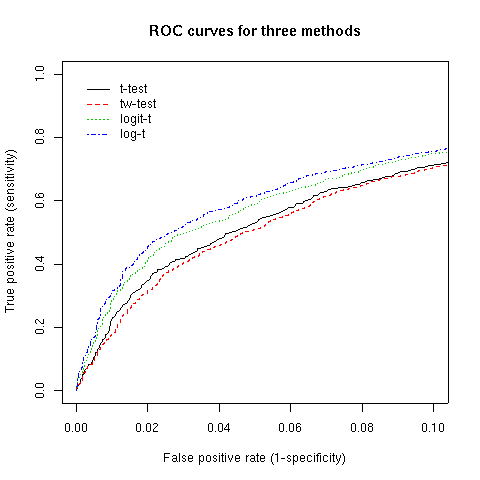

Supplement: Additional File 2 — Similar to the file above, this file contains figures of ROC curves but with data generated from the negative binomial distribution. [file 1471-2105-6-165-S2.gz › ps_fig/2_0.17_0.002.png]

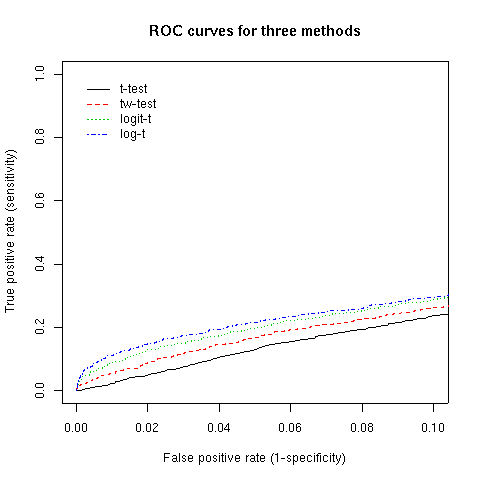

Supplement: Additional File 2 — Similar to the file above, this file contains figures of ROC curves but with data generated from the negative binomial distribution. [file 1471-2105-6-165-S2.gz › ps_fig/2_0.42_2e-05.png]

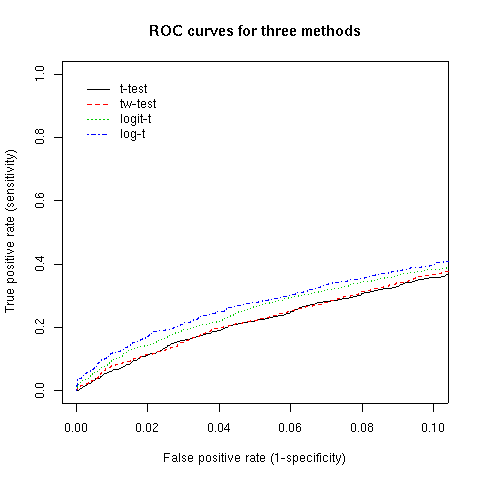

Supplement: Additional File 2 — Similar to the file above, this file contains figures of ROC curves but with data generated from the negative binomial distribution. [file 1471-2105-6-165-S2.gz › ps_fig/2_0.42_0.0001.png]

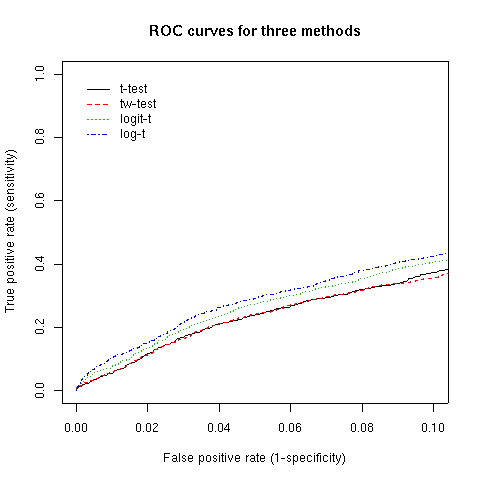

Supplement: Additional File 2 — Similar to the file above, this file contains figures of ROC curves but with data generated from the negative binomial distribution. [file 1471-2105-6-165-S2.gz › ps_fig/2_0.42_0.0002.png]

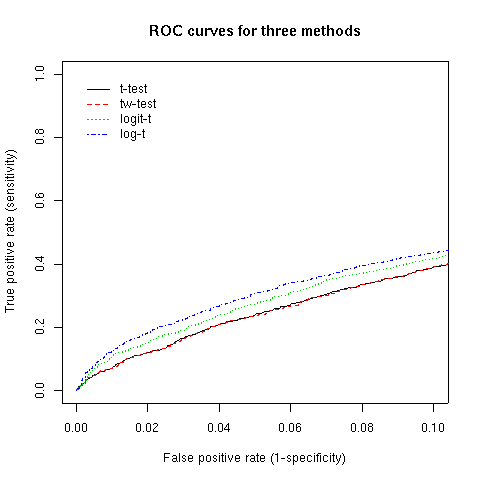

Supplement: Additional File 2 — Similar to the file above, this file contains figures of ROC curves but with data generated from the negative binomial distribution. [file 1471-2105-6-165-S2.gz › ps_fig/2_0.42_0.001.png]

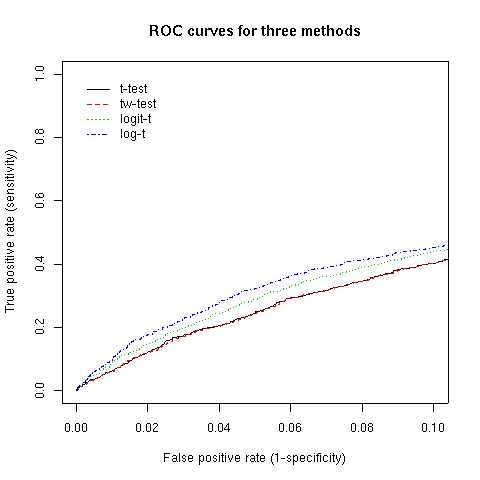

Supplement: Additional File 2 — Similar to the file above, this file contains figures of ROC curves but with data generated from the negative binomial distribution. [file 1471-2105-6-165-S2.gz › ps_fig/2_0.42_0.002.png]

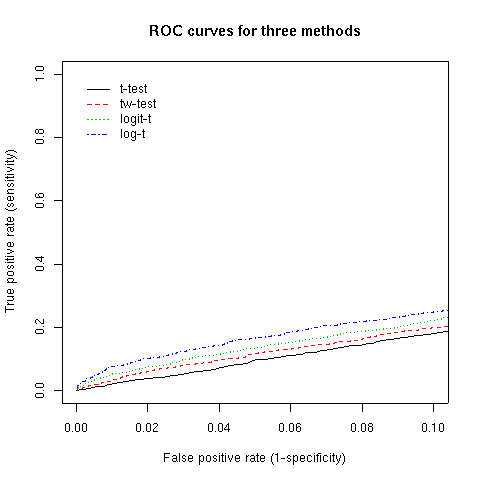

Supplement: Additional File 2 — Similar to the file above, this file contains figures of ROC curves but with data generated from the negative binomial distribution. [file 1471-2105-6-165-S2.gz › ps_fig/2_0.95_2e-05.png]

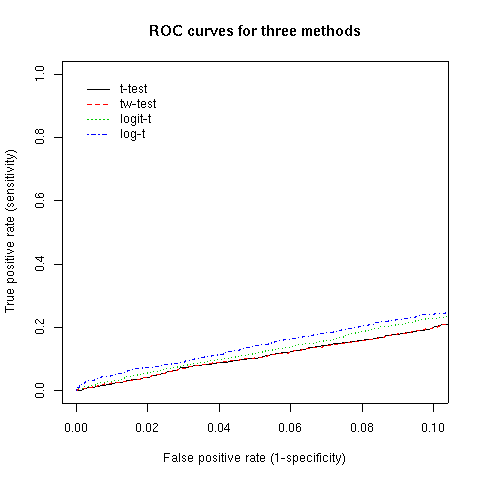

Supplement: Additional File 2 — Similar to the file above, this file contains figures of ROC curves but with data generated from the negative binomial distribution. [file 1471-2105-6-165-S2.gz › ps_fig/2_0.95_0.0001.png]

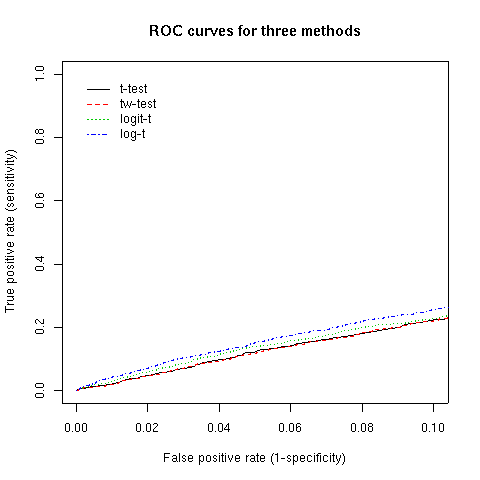

Supplement: Additional File 2 — Similar to the file above, this file contains figures of ROC curves but with data generated from the negative binomial distribution. [file 1471-2105-6-165-S2.gz › ps_fig/2_0.95_0.0002.png]

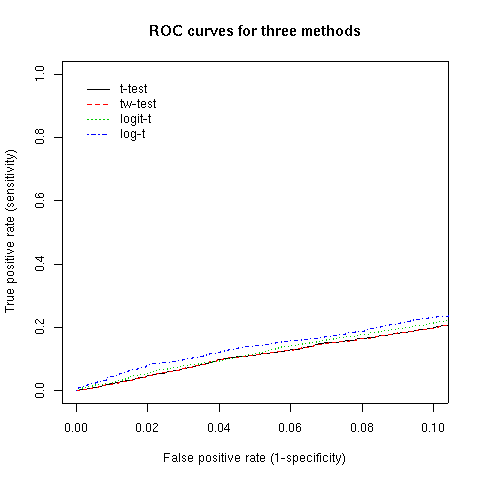

Supplement: Additional File 2 — Similar to the file above, this file contains figures of ROC curves but with data generated from the negative binomial distribution. [file 1471-2105-6-165-S2.gz › ps_fig/2_0.95_0.001.png]

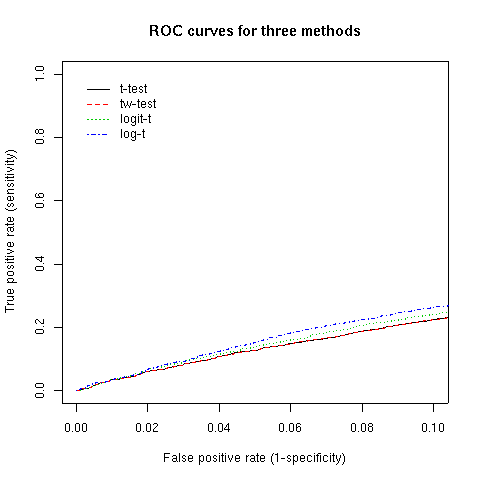

Supplement: Additional File 2 — Similar to the file above, this file contains figures of ROC curves but with data generated from the negative binomial distribution. [file 1471-2105-6-165-S2.gz › ps_fig/2_0.95_0.002.png]

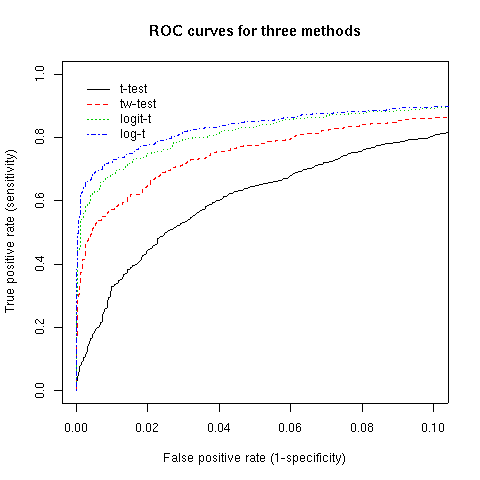

Supplement: Additional File 2 — Similar to the file above, this file contains figures of ROC curves but with data generated from the negative binomial distribution. [file 1471-2105-6-165-S2.gz › ps_fig/4_0.17_2e-05.png]

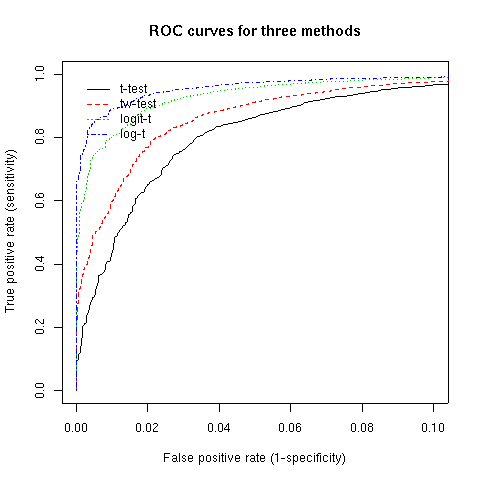

Supplement: Additional File 2 — Similar to the file above, this file contains figures of ROC curves but with data generated from the negative binomial distribution. [file 1471-2105-6-165-S2.gz › ps_fig/4_0.17_0.0001.png]

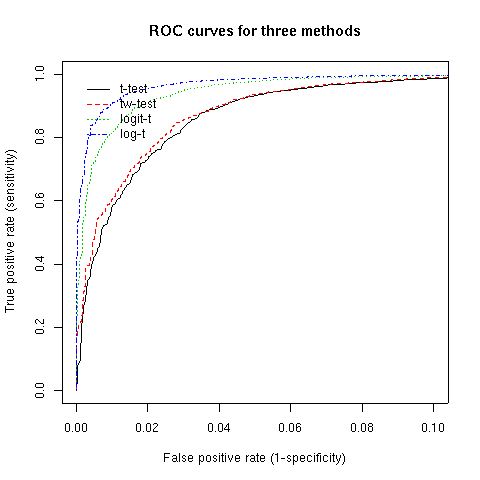

Supplement: Additional File 2 — Similar to the file above, this file contains figures of ROC curves but with data generated from the negative binomial distribution. [file 1471-2105-6-165-S2.gz › ps_fig/4_0.17_0.0002.png]

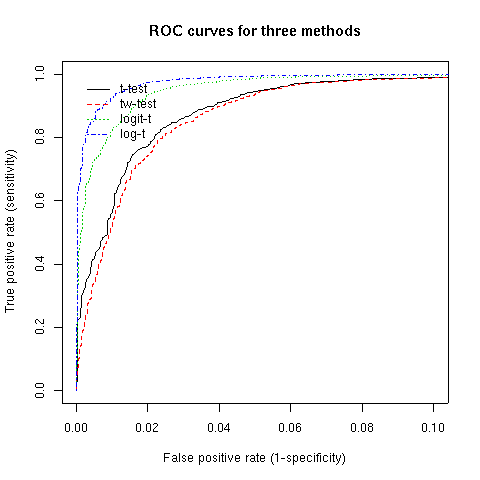

Supplement: Additional File 2 — Similar to the file above, this file contains figures of ROC curves but with data generated from the negative binomial distribution. [file 1471-2105-6-165-S2.gz › ps_fig/4_0.17_0.001.png]

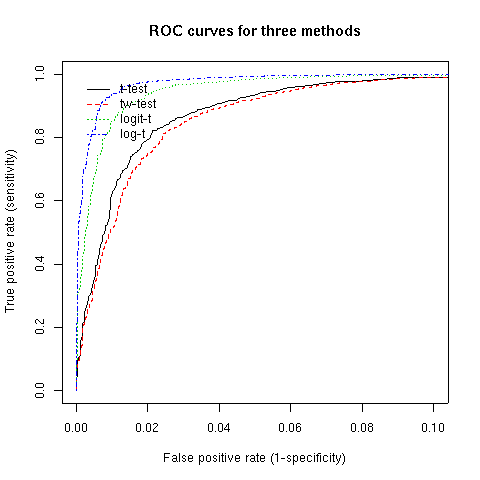

Supplement: Additional File 2 — Similar to the file above, this file contains figures of ROC curves but with data generated from the negative binomial distribution. [file 1471-2105-6-165-S2.gz › ps_fig/4_0.17_0.002.png]

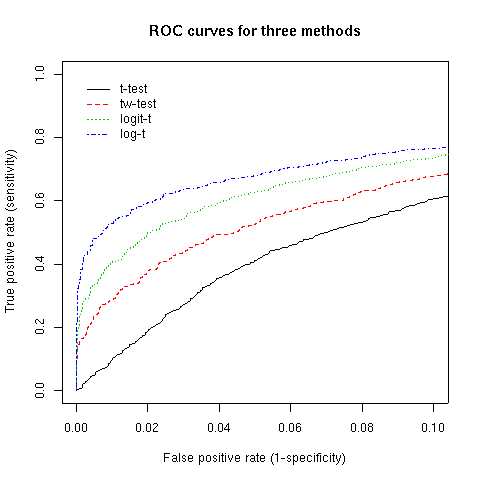

Supplement: Additional File 2 — Similar to the file above, this file contains figures of ROC curves but with data generated from the negative binomial distribution. [file 1471-2105-6-165-S2.gz › ps_fig/4_0.42_2e-05.png]

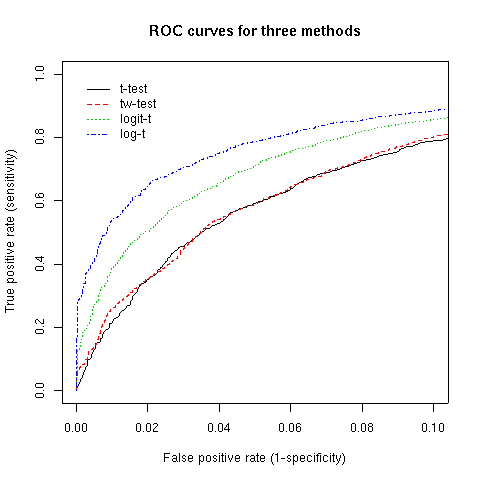

Supplement: Additional File 2 — Similar to the file above, this file contains figures of ROC curves but with data generated from the negative binomial distribution. [file 1471-2105-6-165-S2.gz › ps_fig/4_0.42_0.0001.png]

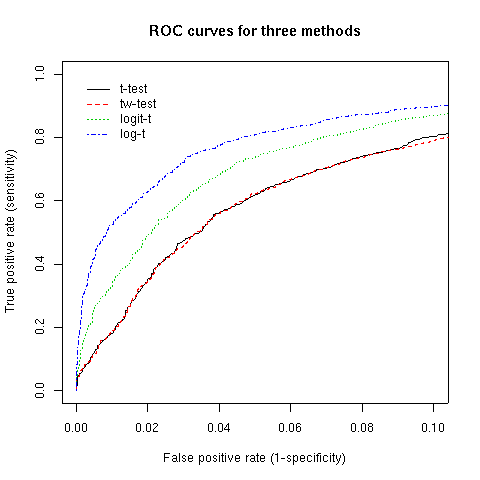

Supplement: Additional File 2 — Similar to the file above, this file contains figures of ROC curves but with data generated from the negative binomial distribution. [file 1471-2105-6-165-S2.gz › ps_fig/4_0.42_0.0002.png]

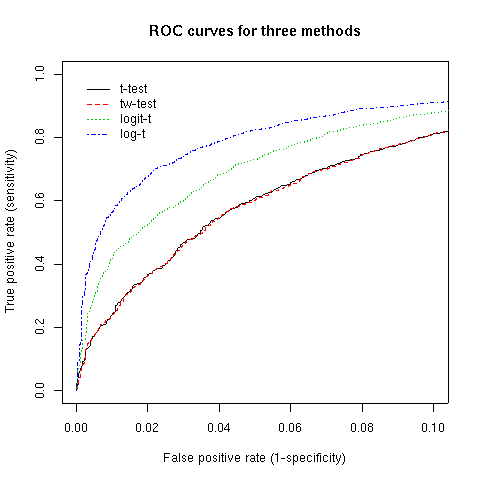

Supplement: Additional File 2 — Similar to the file above, this file contains figures of ROC curves but with data generated from the negative binomial distribution. [file 1471-2105-6-165-S2.gz › ps_fig/4_0.42_0.001.png]

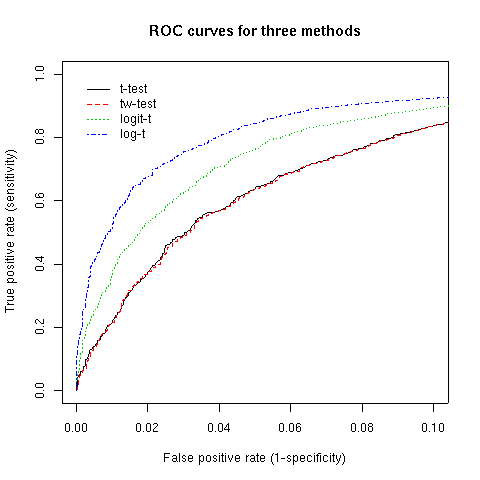

Supplement: Additional File 2 — Similar to the file above, this file contains figures of ROC curves but with data generated from the negative binomial distribution. [file 1471-2105-6-165-S2.gz › ps_fig/4_0.42_0.002.png]

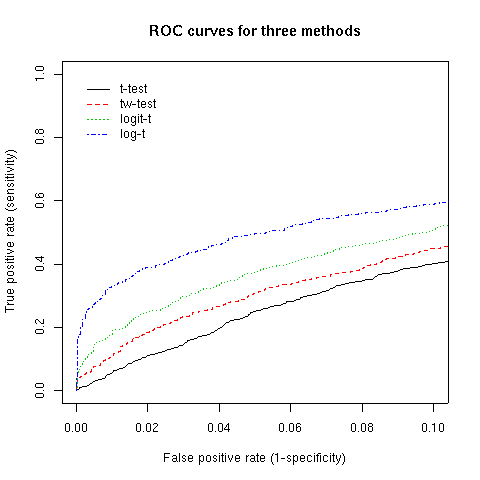

Supplement: Additional File 2 — Similar to the file above, this file contains figures of ROC curves but with data generated from the negative binomial distribution. [file 1471-2105-6-165-S2.gz › ps_fig/4_0.95_2e-05.png]

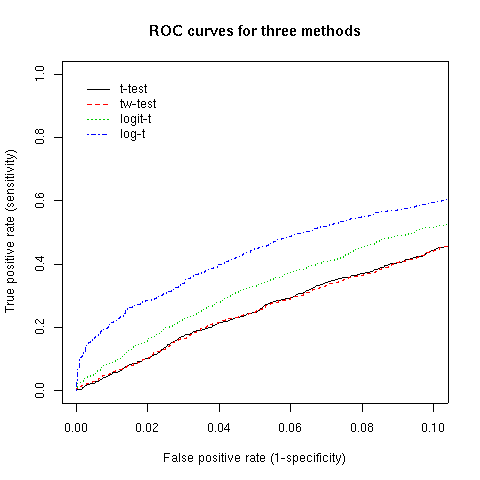

Supplement: Additional File 2 — Similar to the file above, this file contains figures of ROC curves but with data generated from the negative binomial distribution. [file 1471-2105-6-165-S2.gz › ps_fig/4_0.95_0.0001.png]

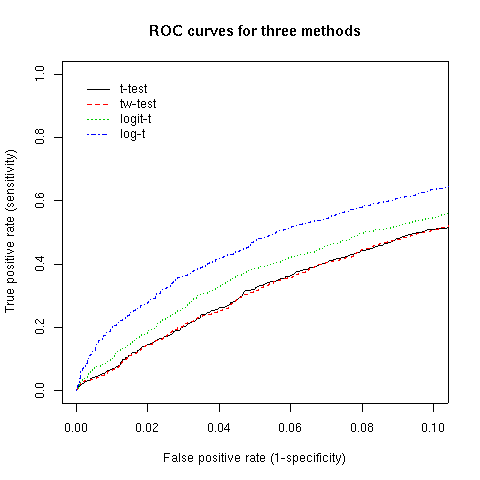

Supplement: Additional File 2 — Similar to the file above, this file contains figures of ROC curves but with data generated from the negative binomial distribution. [file 1471-2105-6-165-S2.gz › ps_fig/4_0.95_0.0002.png]

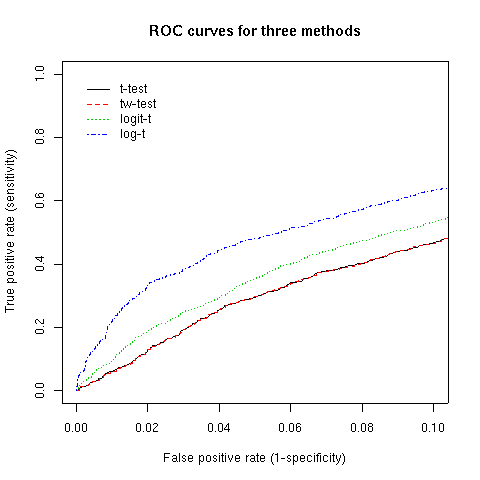

Supplement: Additional File 2 — Similar to the file above, this file contains figures of ROC curves but with data generated from the negative binomial distribution. [file 1471-2105-6-165-S2.gz › ps_fig/4_0.95_0.001.png]

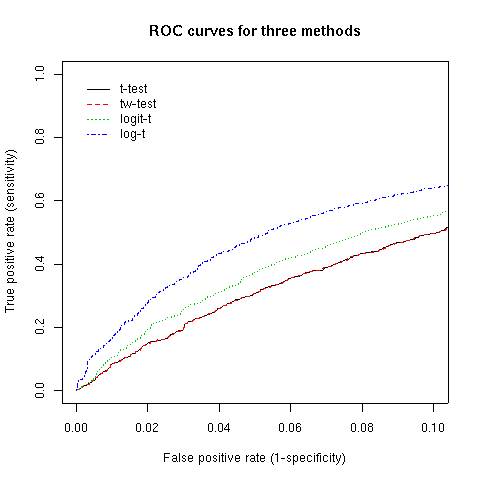

Supplement: Additional File 2 — Similar to the file above, this file contains figures of ROC curves but with data generated from the negative binomial distribution. [file 1471-2105-6-165-S2.gz › ps_fig/4_0.95_0.002.png]
